# Adjusting the catalytic properties of cobalt ferrite nanoparticles by pulsed laser fragmentation in water with defined energy dose

**DOI:** 10.1038/s41598-017-13333-z

**Published:** 2017-10-13

**Authors:** Friedrich Waag, Bilal Gökce, Chakrapani Kalapu, Georg Bendt, Soma Salamon, Joachim Landers, Ulrich Hagemann, Markus Heidelmann, Stephan Schulz, Heiko Wende, Nils Hartmann, Malte Behrens, Stephan Barcikowski

**Affiliations:** 1 University of Duisburg-Essen, Center for Nanointegration Duisburg-Essen (CENIDE), Duisburg, 47057 Germany; 2 University of Duisburg-Essen, Institute of Technical Chemistry I, Essen, 45141 Germany; 3 University of Duisburg-Essen, Institute of Inorganic Chemistry, Essen, 45141 Germany; 4 University of Duisburg-Essen, Faculty of Physics, Duisburg, 47057 Germany; 5 University of Duisburg-Essen, Interdisciplinary Center for Analytics on the Nanoscale (ICAN), Duisburg, 47057 Germany

## Abstract

Highly active, structurally disordered CoFe_2_O_4_/CoO electrocatalysts are synthesized by pulsed laser fragmentation in liquid (PLFL) of a commercial CoFe_2_O_4_ powder dispersed in water. A partial transformation of the CoFe_2_O_4_ educt to CoO is observed and proposed to be a thermal decomposition process induced by the picosecond pulsed laser irradiation. The overpotential in the OER in aqueous alkaline media at 10 mA cm^−2^ is reduced by 23% compared to the educt down to 0.32 V with a Tafel slope of 71 mV dec^−1^. Importantly, the catalytic activity is systematically adjustable by the number of PLFL treatment cycles. The occurrence of thermal melting and decomposition during one PLFL cycle is verified by modelling the laser beam energy distribution within the irradiated colloid volume and comparing the by single particles absorbed part to threshold energies. Thermal decomposition leads to a massive reduction in particle size and crystal transformations towards crystalline CoO and amorphous CoFe_2_O_4_. Subsequently, thermal melting forms multi-phase spherical and network-like particles. Additionally, Fe-based layered double hydroxides at higher process cycle repetitions emerge as a byproduct. The results show that PLFL is a promising method that allows modification of the structural order in oxides and thus access to catalytically interesting materials.

## Introduction

Hydrogen is probably the cleanest fuel on earth, as it can be gained from water and reacts to water within its consumption. Nowadays, steam reforming of carbon-based fuels represents the main source of H_2_, but mainly fossil fuels are used and the carbon contamination of the produced H_2_ is high^1^. The competing technology of electrolysis of water, which generates pure H_2_, is still consuming too much energy to implement as a long-term solution for the covering of our energy demand^2^. This is mainly due to the sluggish kinetics of the oxygen evolution reaction because of its four electron transfer, which requires suitable catalytic materials. Thus, the design of a highly active and cost-effective catalyst, to reduce the overpotential of water electrolysis, will play a key role in future’s success of H_2_ as fuel. First row transition metals probably represent the most promising class of materials because of their abundance in earth’s crust and proven activity in the OER in alkaline media^3–11^. However, their activity is usually lower compared to that of scarce and nobler materials like RuO_2_ or IrO_2_. Thus, finding a suitable way to improve their OER activity is highly desirable. In general, it is believed that many heterogeneous catalysts can be further optimized by increasing the abundance of sites with high surface energy that are often related to surface defects. Thus, increasing the structural disorder of oxide particles seems promising, but a true synthetic control of metastable and defective nanoparticles is difficult to obtain as only a limited number of synthesis methods are adequate for this purpose.

Among these, laser-induced transformations of particle morphology, crystallinity and chemical composition are promising and may lead to superior electrochemical properties. In this study, we focus on a catalyst synthesis via pulsed laser fragmentation in liquid (PLFL) approach that is applied to treat commercial CoFe_2_O_4_ powder with the aim to induce structural disorder and improve its OER activity. CoFe_2_O_4_ is a spinel with interesting electronic and magnetic properties^12–14^, and is known to exhibit OER activity, but typically cannot compete with the best noble metal-based electrodes. Nevertheless, the research interest in spinels and especially ferrites, also perovskite-type, for catalytic applications is high. Taffa *et al*. recently published a mini-review on spinel-type ferrites as catalysts for photoelectrochemical water splitting^15^. Another short review on ferrites in more general photocatalytic applications and on their synthesis has been published by Casbeer *et al*. in 2012^16^. As well focused on photocatalysis, water splitting and pollutant decomposition, is the book chapter on ferrites of Ren *et al*.^17^. Kharisov *et al*. included all fields of hetrogeneous catalysis into their mini-reviev on ferrite nanoparticle catalysts of 2014^18^. In regard to our study, the up to date and extensive review of Zhao *et al*. on spinels as catalysts for OER and ORR, that also contains a detailed part on preparation methods, is of highest relevance^19^.

Importantly, we utilize a flowing, not enclosed liquid jet for PLFL of CoFe_2_O_4_ dispersed in water^20^. Advantages of this setup include its scalability, precise energy dose quantification^21^, and higher intensity input into the colloid since no windows are required. This method allows precise control of oxide properties by applying the PLFL semi-continuously as shown by Lau *et al*. who were able to tune the band gap energy of ZnO particles^21^.

Pulsed laser ablation in liquid (PLAL)^22–24^ and PLFL^25–27^ are two methods that currently attract interest as synthesis routes for nanoscale catalysts^28^. These methods lead, due to the absence of ligands and precursor residues, to nanoparticles with bare surfaces^29^. This enables increased interaction between catalyst and adsorptive which makes nanoparticles prepared by PLAL or PLFL suitable for heterogeneous catalysis. For instance, Blakemore *et al*. fabricated the most active unsupported spinel-type cobalt oxide nanocatalyst for OER, published to that time, by a PLFL of Co microscale powder in water^30^. The OER activity of the best CoFe_2_O_4_/CoO catalysts in this study is higher than that reported previously by McCrory *et al*. for a CoFeO_x_ catalyst^11^. We reach an overpotential of 0.32 mV at 10 mA cm^−2^ in 1 M KOH compared to that of 0.37 mV in 1 M NaOH reached by McCrory *et al*.

## Results and Discussion

PLFL is performed in a semi-continuous liquid flow^21,31^. We arrange the flow as a free – not enclosed – jet that is perpendicularly crossing the laser beam. In the following sections, we use the term *passage* (p in figure legends) as a description for the complete colloid volume flowing through the laser beam one time. Furthermore, we define the *specific energy dose* here as the energy, which is extinct by the total volume of particulate matter of the colloid within one passage. A definition of a volume related dose is reasonable since we compare the light absorption properties of particles of different sizes to thermal transformation thresholds.

### UV-vis extinction spectroscopy

**Figure 1:**
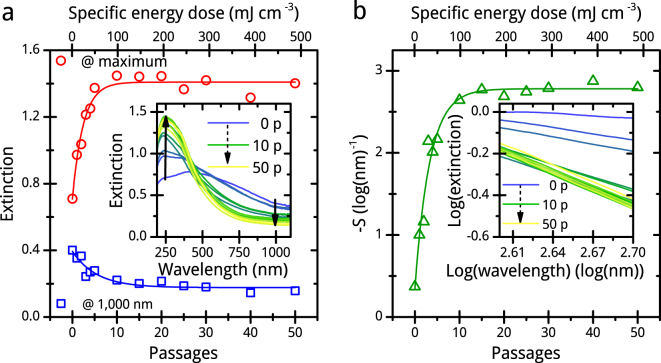
UV-vis extinction data of chosen CoFe_2_O_4_ samples. Change of extinction of products after different numbers of PLFL passages (p) of CoFe_2_O_4_ in water at 1,000 nm and maximum (**a**) and negative Furlong slopes (−*S*) (**b**) in dependence to PLFL passages or the specific energy dose, respectively. The inlet of (**a**) shows the corresponding spectra of in (**a**) plotted extinction data, and that of (**b**) the double logarithmic plot of these spectra in the blue-UV spectral range.

We observe and detect by UV-vis extinction spectroscopy a change of the colloids light extinction properties with increasing number of PLFL passages or specific energy dose, which indicates morphology transitions of the CoFe_2_O_4_ educt during PLFL (Fig. 1). The extinction of the colloid decreases for red and infrared wavelengths while it increases for blue and ultraviolet wavelengths, a typical effect induced by size dependent particle scattering (Fig. 1a)^32,33^. Furlong *et al.* implemented a simple tool for presenting this effect by double logarithmic plotting the negative slope of the blue extinction shoulder (Fig. 1b)^34^. In our case, the so-called Furlong-plot reveals that there is a saturation in the extinction change of the product after the tenth passage of PLFL.

### High-resolution transmission electron microscopy (HR-TEM)

**Figure 2:**
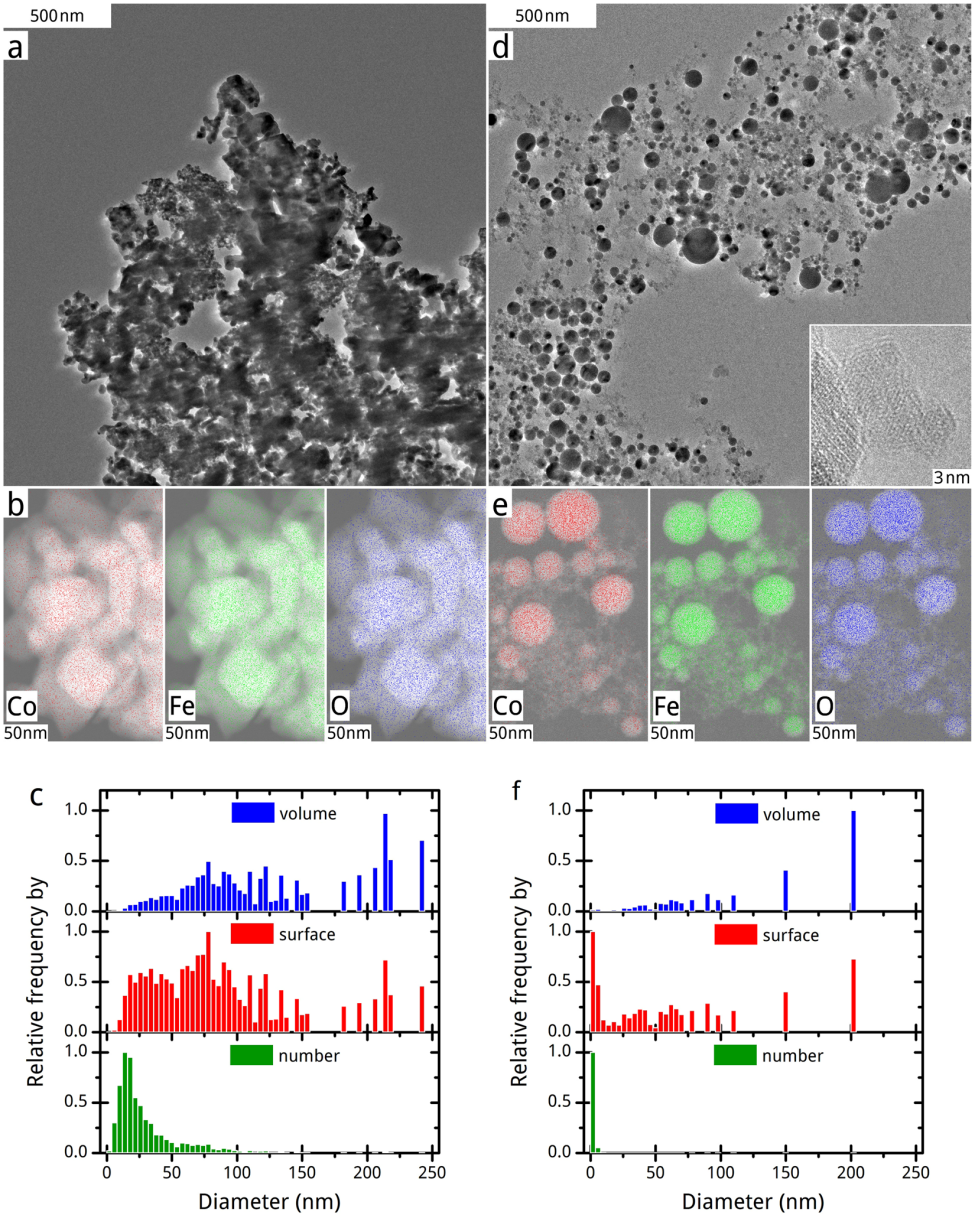
TEM and EDX investigation of chosen CoFe_2_O_4_ samples. HR-TEM micrographs (**a**,**d**), EDX elemental mappings (**b**,**e**) and size histograms (**c**,**f**) of the CoFe_2_O_4_ educt on the left (**a** to **c**) and the sample after tenth PLFL passage on the right (**d** to **f**). The inlet of HR-TEM micrograph of the product (**d**) shows a conglomerate of particles of the by number dominant size fraction.

We perform a detailed investigation of the particle morphology evolution in dependence to the passage number or specific energy dose by use of HR-TEM. Figure 2a,d shows representative micrographs of the educt and the sample after the tenth passage, as well as particle size histograms and EDX elemental data. Obviously, mainly spherical particles form during PLFL and their aggregation on the TEM grid seems to be lowered compared to the educt particles. At a higher magnification, as shown in Fig. 2d, a fraction of ultra-small amorphous particles with diameters around 2 nm appears. This particle size fraction is dominant in number (Fig. 2f). It fills up the space between the bigger spherical particles and generates a seemingly higher dispersity of product particles. Nevertheless, the contribution of bigger (>4 nm) particles to the total product surface is still higher in sum. We present additional HR-TEM micrographs and size histograms for products after other passage numbers in Supplementary Fig. S1.

**Figure 3:**
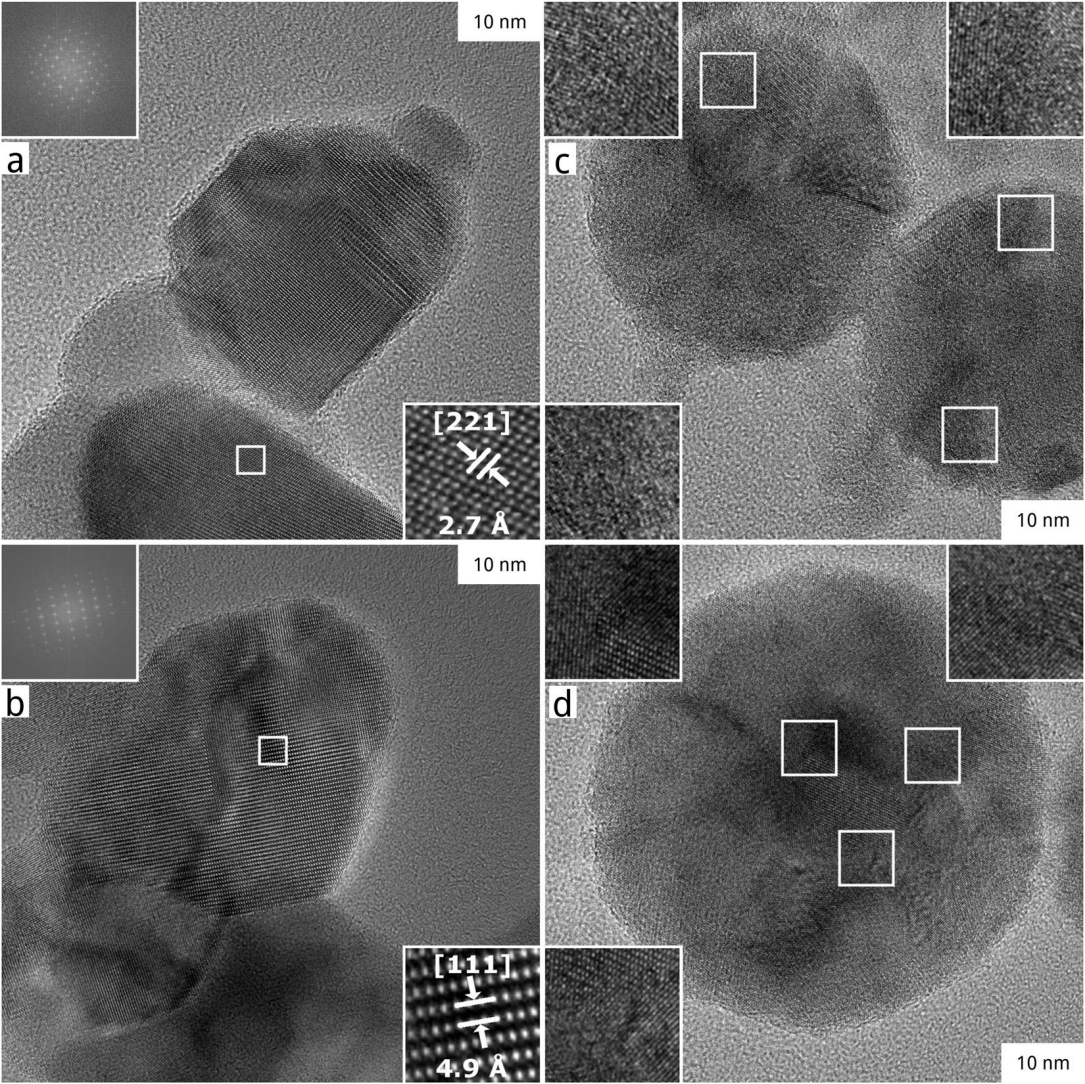
Crystal lattice investigation of single particles of chosen CoFe_2_O_4_ samples. Micrographs of representative particles of the CoFe_2_O_4_ educt on the left (**a**,**b**) and the product after the tenth passage of PLFL on the right (**c**,**d**). The inlays of educt’s micrographs show reciprocal and real single crystal lattice structures with typical plane distances of CoFe_2_O_4_. In product’s micrographs, all inlays show magnifications of multi-crystalline structures within the particles.

Since morphology changes are drastic and at least in part thermally induced, as indicated by the spherical particle morphology, a possible thermochemical decomposition is investigated by energy dispersive X-ray spectroscopy (EDX). The occurrence of such thermal effects during PLFL depends on the relation of the relaxation time of the electron-phonon coupling of the irradiated material to the laser pulse length. A pulse length longer than the relaxation time enables a transfer of by electrons absorbed laser light energy to the lattice atoms. The relaxation time of CoFe_2_O_4_ or that of comparable Fe_3_O_4_ is unknown, but Chen *et al*. observed lattice heating of Fe_3_O_4_ nanocrystals irradiated by 60 fs pulses^35^. Conclusively, an occurrence of thermal material transition effects is likely at our applied laser pulse length of 10 ps for CoFe_2_O_4_ nanocrystals. As we illustrate in Fig. 2, there is no significant change in the elemental composition during PLFL. Co, Fe and O are homogenously distributed in the different particle size fractions. However, we find single particles with diameters bigger than 10 nm – approximately each twentieth – that show a much stronger signal for Co in relation to Fe than expected for CoFe_2_O_4_. One of those particles, with a diameter of around 50 nm, is shown in the top mid of Fig. 2e. This single particle has an atomic ratio of Co and Fe of nearly 1:1 instead of 1:2 as assumed for CoFe_2_O_4_. This significant deviation of this byproduct particle refers to a possible thermal decomposition of CoFe_2_O_4_ during PLFL that needs to go along with a change in particles lattice structure and a release of Fe species to the liquid. A comparison of the lattice structures of representative educt and product (after tenth passage) particles of around 50 nm in diameter is shown in Fig. 3. The mainly single-crystalline lattice structure of educt particles (Fig. 3a,b), clearly identifiable as CoFe_2_O_4_, converts into a multi-crystalline one (Fig. 3c,d), whose single domains could not be further investigated by fast Fourier transformation due to lattice disorder and overlay. Thus, it remains unclear from HR-TEM combined with EDX analysis which and to what extent Co-rich phases form during PLFL. Furthermore, a Fe-rich phase should also appear conclusively beside a Co-rich one. We proceed further analytics: X-ray diffraction (XRD), X-ray photoelectron spectroscopy (XPS) as well as Mössbauer spectroscopy, to identify Co- and Fe-rich phases in the products after different numbers of PLFL passages.

### Powder X-ray diffraction (PXRD) analysis

**Figure 4:**
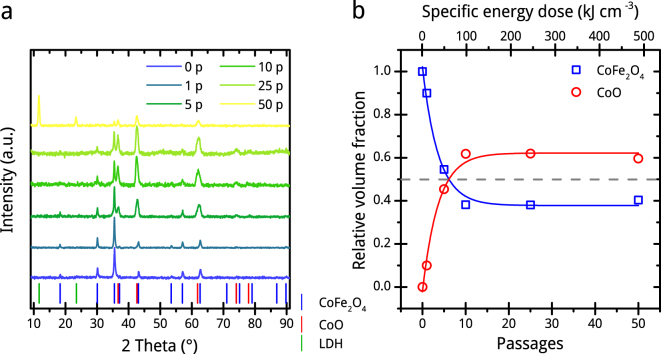
Crystal lattice investigation of chosen CoFe_2_O_4_ samples. PXRD patterns (**a**) and phase composition by volume derived from Rietveld refinement (**b**) of educt (0 p) and products after different numbers of PLFL passages (1 p, 5 p, 10 p, 25 p and 50 p). The diagram of PXRD patterns shows the positions of diffraction peaks of pure phases of CoFe_2_O_4_, CoO and main diffraction peaks of fougerite (LDH) in addition.

To clarify the occurrence of a probable thermal decomposition of CoFe_2_O_4_ during picosecond pulsed laser irradiation, we perform PXRD measurements of products after different passages. Figure 4 shows the background corrected and normalized powder diffractograms, which clearly demonstrate two emerging phases beside CoFe_2_O_4_: CoO significantly present directly after the first passage on the one side and a layered double hydroxide (LDH) after 50^th^ passage on the other. The relative amount of LDH in this sample is 18.6 vol.-%. This observation confirms the theory of the formation of Co-rich particles by a thermal decomposition, during which iron species are released to the aqueous phase and organize to LDH during further laser processing. Hunter *et al*. also observed a formation of LDH during PLAL of Fe and Ni foils immersed in alkaline metal salt solutions^36^. (The LDH formation is further described in the supplementary information). The amount by volume of CoO relative to CoFe_2_O_4_ increases logarithmically with passage numbers and reaches saturation in 0.61 after tenth the passage (Fig. 4b). This lattice transformation correlates well with the change in UV-vis extinction properties of the colloid and at least partly with the ratio of the ultra-small particle fraction, which is the dominant fraction by surface after the tenth passage of PLFL (Supplementary Fig. S1). If we assume this fraction to form through a thermal decomposition process, the lattice transformation should also be caused by this decomposition with a high probability. To further investigate this effect, we applied the PLFL at same conditions for ten passages to a CoO submicron powder, and it fully transformed to Co_3_O_4_ (Fig. S4). The spinel seems to be the preferred crystalline phase under the experimental conditions. This possibly explains why there is not a full transformation of CoFe_2_O_4_ to CoO, but not why CoO forms at all. Conclusively, the formation of CoO depends on the presence of Fe^3+^ and absence of Co^3+^ in CoFe_2_O_4_. Both phases, CoO and CoFe_2_O_4_, are expected to coexist in single particles since we could find Co-rich but no Fe-free particles via TEM-EDX.

### X-ray photoelectron spectroscopy (XPS)

**Figure 5:**
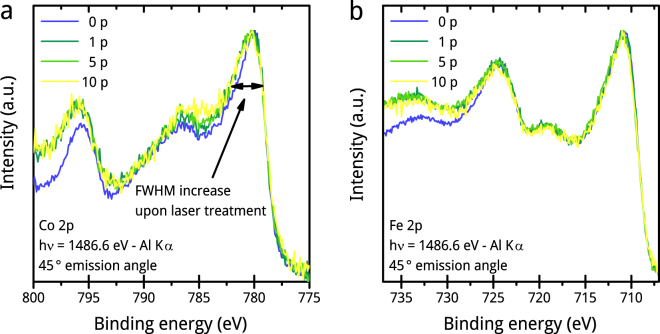
XPS spectra of chosen CoFe_2_O_4_ samples. Co 2p (**a**) and Fe 2p (**b**) XPS spectra of the educt (0 p) and after first (1 p), fifth (5 p) and tenth (10 p) PLFL passages. The spectra show that both metals are completely oxidized. The best result in peak fitting is achieved when using a mixture of small amounts of the oxides, CoO and Fe_2_O_3_, with the hydroxide species, namely Co(OH)_2_ and FeOOH. Due to the low signal to noise ratio an oxide to hydroxide ratio cannot be calculated.

We carry out XPS measurements to further investigate the change of oxidation states of Co and Fe during PLFL. Figure 5 shows the Co 2p and Fe 2p signals from XPS measurements on the CoFe_2_O_4_ powders after different numbers of passages. Both spectra are best described by a mixture of hydroxide and oxide species, with the latter being less pronounced^37^. The quality of the signal, however, does not allow any quantification in this regard.

We find no strong changes in the oxidation state both for Fe and Co upon PLFL passage. In the case of Co, we identify an increase in FWHM of the main Co 2p_3/2_ peak at around 780 eV after the first PLFL passage. This is a weak hint that the oxide/hydroxide ratio possibly changes^37^. The amount of secondary electrons increases in both spectra, Co and Fe, upon PLFL passage. This possibly hints to a higher disordering in the particles crystal structure. A higher disordering is expected due to the formation of multi-crystalline particles, that can be observed by HR-TEM (Fig. 3), and the partial lattice transformation to CoO, clearly shown by PXRD diffractograms (Fig. 4).

### Mössbauer spectroscopy

**Figure 6:**
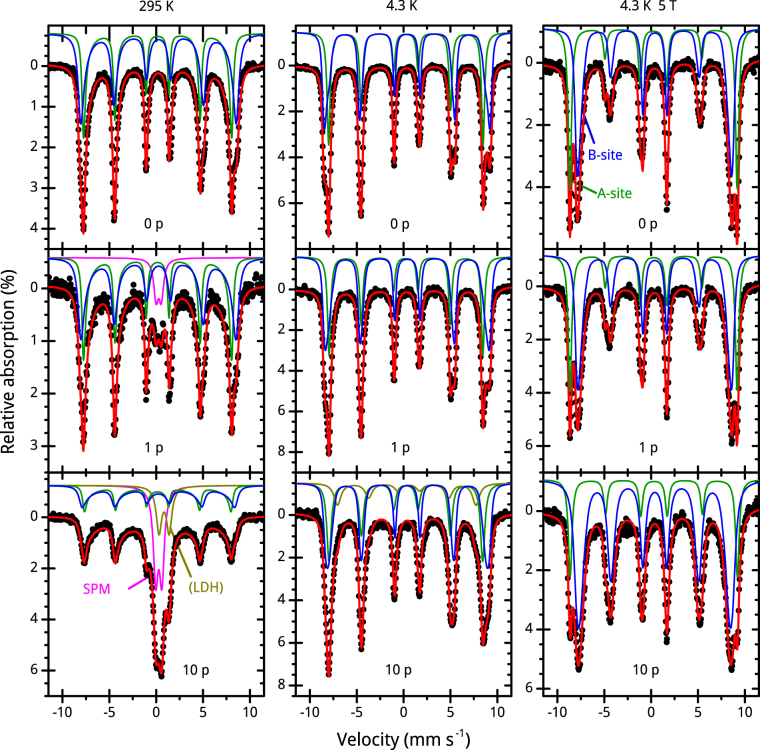
Mössbauer spectra of chosen CoFe_2_O_4_ samples. Mössbauer spectra of CoFe_2_O_4_ educt (0 p) and products after first (1 p) and tenth (10 p) PLFL passage measured at room temperature (left), 4.3 K (center) and 4.3 K with an applied field of 5 T (right). The subspectra correspond to: Tetrahedral A-sites (green) and octahedral B-sites (blue) of CoFe_2_O_4_, superparamagnetic CoFe_2_O_4_ nanoparticles (magenta) and a presumable parasitic LDH phase (dark yellow).

We record Mössbauer spectra of the CoFe_2_O_4_ educt and products after the first and tenth PLFL passage at room temperature (left), 4.3 K (centre) and at 4.3 K with an applied magnetic field of 5 T along the γ-ray propagation direction (right), shown in Fig. 6. For the educt, the 5 T spectrum shows clearly resolved sextet subspectra for (tetrahedral) A- and (octahedral) B-sites occupied by the Fe-ions, with hyperfine parameters that are typical for CoFe_2_O_4_^38,39^. Two hyperfine field distributions are used to reproduce the fine structure of the subspectra, taking into account the correlation between the effective magnetic field and the spin canting angle. We determine the inversion parameter, calculated from the ratio of the two relative spectral areas, to be 0.79(2) while the mean canting angles are 16(1) ° and 30(1) ° for A- and B-sites, respectively. Up to room temperature, the educt remains magnetically blocked, displaying only minor traces of beginning superparamagnetic relaxation, revealed by the asymmetric inner shoulders of lines 1 and 6, which are reproduced theoretically using the many state relaxation model by Jones and Srivastava^40^. The product after the first PLFL passage shows similar behaviour at low temperatures as the educt, while at room temperature, a small superparamagnetic doublet is visible, with a relative spectral area of 7.7(2) %. This indicates a decrease in particle size, as we observe it in the HR-TEM micrographs in Supplementary Fig. S1. The inversion parameter decreases slightly to 0.74(2), while the A- and B-site canting angles increase to 18(1) ° and 33(1) °, respectively.

We observe the largest changes for the product after the tenth PLFL passage, with the 5 T spectrum containing broader absorption lines, possibly caused by a static hyperfine field distribution due to disorder effects. Spin canting angles on A- and B-sites increased strongly to 26(1) ° and 43(1) °, prohibiting a precise determination of the relative spectral areas and, therefore, of the inversion parameter. The strong increase in spin canting is indicative of a higher surface contribution, corresponding to a smaller average particle diameter, as supported by HR-TEM images (Fig. 2 and Supplementary Fig. S1). Alternatively, distinct spin canting can also be caused by a high degree of magnetic disorder, originating from structural defects induced by the laser treatment, or a combination of both effects. At room temperature, the spectral area of the superparamagnetic doublet is strongly increased to 27.1(4) % relative to 7.7(2) % in the product after first passage. This increase in superparamagnetic fraction is in agreement with the appearance of ultra-small particles visible in the HR-TEM analysis of the products after first and tenth passage of PLFL (Supplementary Fig. S1). We see an additional subspectrum with ca. 15% relative spectral area in the centre of the spectrum, which can be reproduced by a doublet (*δ* = 0.95(1) mm s^−1^ rel. to α-Fe, Δ*E*_*Q*_ = 1.09(1) mm s^−1^) and can be assigned to an additional parasitic compound, presumably the LDH identified in PXRD data (Fig. 4b) and HR-TEM images (Supplementary Fig. S5). Apparently, this phase is magnetically ordered at 4.3 K, as the spectrum at this temperature does not contain any doublet phase, but shows an additional contribution with matching ca. 15% spectral area, with a smaller hyperfine magnetic field compared to the subspectra of CoFe_2_O_4_. The transition from a magnetically ordered to a paramagnetic state at room temperature would match the low Néel temperatures known for Fe-hydroxides.

In summary, the Mössbauer spectroscopy results support our observations within the HR-TEM analysis. The amount of ultra-small, superparamagnetic particles increases with increasing PLFL passage number. Structural disorder is strongly enhanced in the product after the tenth passage, possibly originating from multi-crystalline particles (Fig. 3c,d). Furthermore, a subspectrum, which confirms the presence of LDH, appears in the spectrum recorded at room temperature of the product after the tenth passage.

### Electrochemical analysis

**Figure 7:**
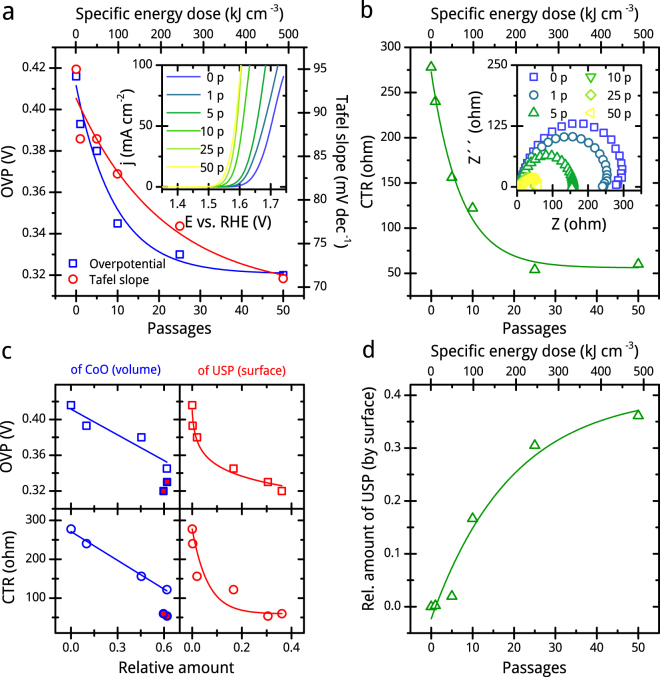
Electrochemical analysis of chosen CoFe_2_O_4_ samples. Overpotentials (OVP) and Tafel slopes including the linear sweep voltammograms as inlet recorded at a sweep of 5 mV s^−1^ in 1 M KOH (**a**), charge transfer resistance (CTR) including Nyquist plots recorded at an overpotential of 350 mV for the educt and products after fifth (5 p), tenth (10 p), 25^th^ (25 p) and 50^th^ (50 p) PLFL passage (**b**), the dependencies of the OVP and CTR on the amount of CoO by volume and of ultra-small particles (USP) by surface (**c**), as well as the change of the relative surface-weighted amount of USP with PLFL passages (**d**). (Red filled data points are not taken into account for fitting).

We investigate the dependence of the electrocatalytic activity of PLFL products for OER in 1 M KOH solution on the specific energy dose by using a three-electrode setup with similar loading of 0.15 mg cm^−2^ at a scan rate of 5 mV sec^−1^. Figure 7a shows the catalytic activity for OER that improves with repetitions of PLFL passages. We use the overpotential to deliver a 10 mA cm^−2^ current density, interesting for solar fuel production, as a convenient figure of merit to evaluate the OER activity^11^. The main graph of Fig. 7a demonstrates the lowering of the overpotential in dependence to the passage number or specific energy dose. The lowest overpotential is reached for the sample after the 50^th^ passage of PLFL with 0.32 V, which is the smallest compared to that after the 25^th^ (0.33 V), tenth (0.345 V), fifth (0.38 V) and first (0.393 V) passage, respectively. In comparison, the educt has an overpotential of 0.416 V. The lowering of the overpotential with increasing number of PLFL passages is in good agreement with the change of UV-vis extinction properties and increasing amount of CoO and ultra-small particles as well. As hydroxides are also known as promising OER catalysts, we expect another positive effect on the activity by the present LDH species^41,42^. Additionally, structural disorder is assumed to enhance further with passage repetition.

We calculate the Tafel slopes as they exhibit the electron transport kinetics. Slopes are collected from fitting polarization data to the Tafel equation (*η* = *a* + *b* log(*j)*, *η* is the overpotential, *b* is the Tafel slope, and *j* is the current density). A linear dependency of *η* vs. log(*j*) is present for all catalysts but we observe different slopes. The product after the 50^th^ passage of PLFL exhibits the lowest Tafel slope of 71 mV dec^−1^ as compared to that after the 25^th^ (77 mV dec^−1^), tenth (83 mV dec^−1^), fifth (87 mV dec^−1^) passage) and first (87 mV dec^−1^), as shown in Fig. 7a. The slope of the educt is 95 mV dec^−1^. Lower Tafel slopes indicate the favourable electron transport that increases OER activity. The improved activity may be caused by enhanced structural disorder and the intrinsic electronic conductivity. To examine the electrode kinetics of the catalytic processes, we perform electrical impedance spectroscopy (EIS). We obtain the charge transfer resistance *R*_*ct*_ by data fitting of Nyquist plot using Randles circuit, recorded at an overpotential of 0.35 V. The Nyquist plot shows semi-circle in high frequency range mainly associated with charge transfer resistance. The diameter of the semi-circle and consequently *R*_*ct*_ decreases with increasing passage number while the products after the 25^th^ and 50^th^ passage exhibit the lowest *R*_*ct*_ of around 55 ohm (Fig. 7b). At tenth passage, R_ct_ is around 125 ohm.

The change of *R*_*ct*_, as well as that of the overpotential and Tafel slopes, correlates in a similar exponential decrease to the passage number or the specific energy dose. However, the correlation is different from that of the crystalline CoO formation, since the formation is already saturated at the tenth passage. This is not the case for the electrochemical properties. When we plot the overpotential and charge transfer resistance versus the volume amount of crystalline CoO (Fig. 7c), a linear dependency can be found until an amount of 0.6 is reached for the first time for the product after the tenth passage of PLFL. For the products after the 25^th^ and 50^th^ passage, we observe a clear gap in the graphs. Conclusively, the CoO formation obviously affects the electrochemical properties, but there is also another contribution. By plotting the electrochemical properties versus the surface-weighted relative amount of ultra-small particles (Fig. 7c), an exponential decaying trend can be observed here without a clear deviation of values of the products after the 25^th^ and 50^th^ passage. But the relative amount of the ultra-small particle fraction is also not saturating after the tenth passage of PLFL (Fig. 7d). A contribution to the activity increase of all products can be expected for the ultra-small particles. Additionally, a strong and trending increase in the BET surface is not observed, as we discuss more in detail in the supplementary information. Thus, we expect the overall improvement of the catalyst’s electrochemical properties during PLFL to mainly originate from structural disorder within the particles in form of crystal separation (up to the product after the tenth passage), multi-crystallinity and amorphousness. In addition, a further contribution on the improvement of the electrochemical properties of the products after the 25^th^ and 50^th^ passage is obvious. Since laser-generated LDH is already known for a good OER performance in the alkaline^36^ and noticeably present in those samples, we assume it to be the additional origin of activity. The results conclusively show that the enhanced catalytic activity originates from not just one laser-induced material property change. Further data of additional experiments for finding more details on the mechanism of activity improvement is presented in the supplementary information. However, we are able to control the catalytic activity of our PLFL products and to lower the overpotential by almost 100 mV by the applied energy dose.

### Pulsed laser fragmentation in liquid (PLFL)

The analytical results refer to laser-induced thermal mechanisms causing at least partly the observed morphology changes, structural disorder in particles’ atomic lattices and decomposition of spinel’s chemical nature. Chen *et al*. have shown that thermal lattice heating occurs during ultrashort laser pulse irradiation of transition metal spinels for Fe_3_O_4_^35^. They induced a lattice heating in Fe_3_O_4_ nanocrystals by applying only 60 fs long laser pulses; our applied pulse length is more than 165 times longer. We describe the pathways of the expected decomposition mechanism in detail in the supplementary information.

**Figure 8:**
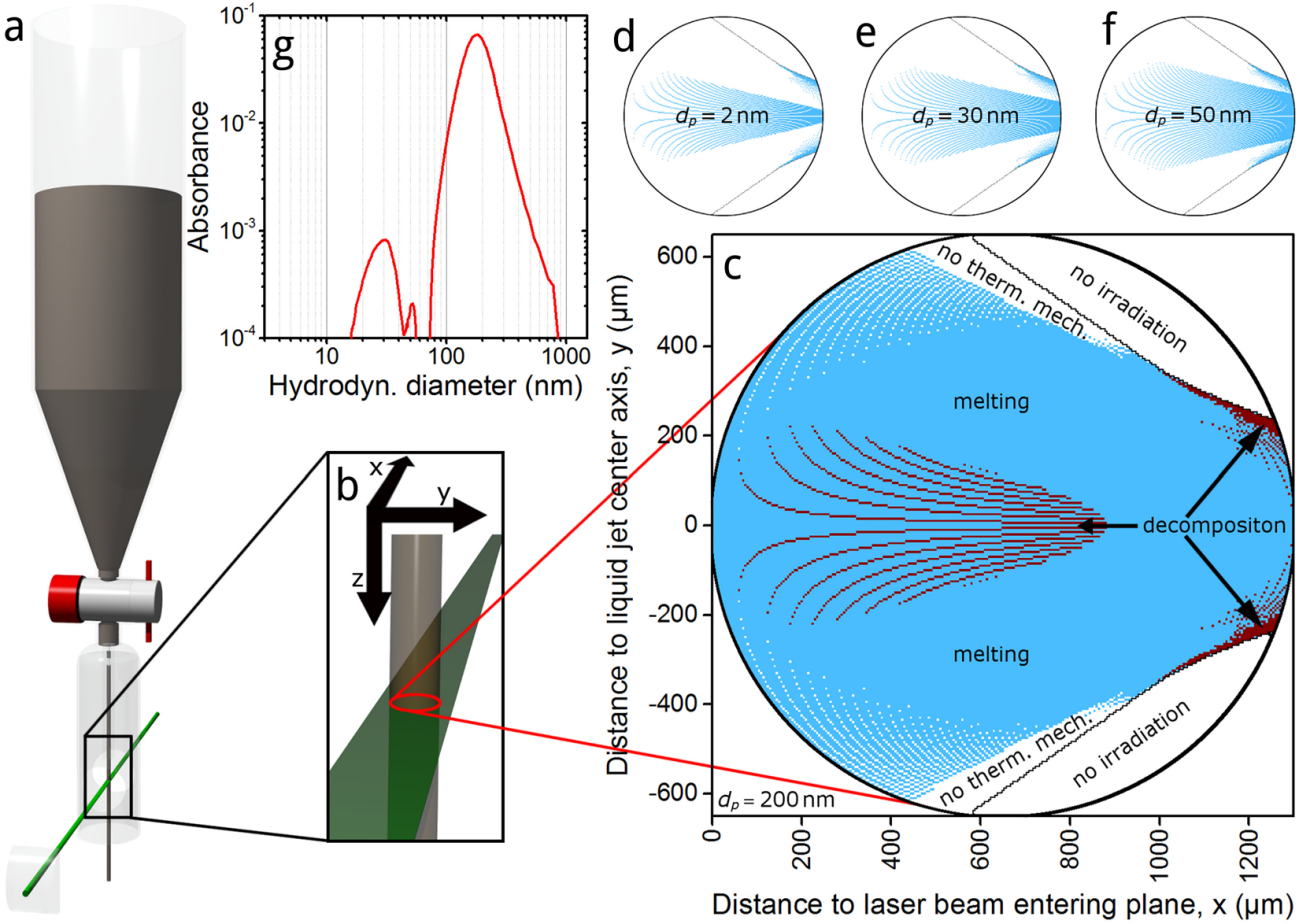
PLFL setup and process modelling results. Illustration of the experimental set-up (**a**,**b**) and thermal mechanism regimes for chosen particle diameters (d_p_) within the liquid jet (**c** to **f**), based on the pulse energy distribution modeling of the liquid jet and calculated particles’ Mie absorption cross-section. The laser beam enters at the left side for diagrams of thermal mechanism regimes. Blue colored regimes relate to melting and red colored ones to decomposition processes. White colored areas within the black circled jet boarder mark either, due to refraction, non-irradiated areas or regimes of such low energy densities that no thermal mechanism affecting a whole particle volume is taking place. In addition, particle diameter dependent absorbance, measured with an analytical disc centrifuge at 405 nm, of the educt powder is shown (**g**) and used for identifying the most absorbing particle size fractions.

Based on the adjusted laser parameter set, the measured laser power and the material properties of the CoFe_2_O_4_ educt colloid, we investigate the lateral distribution of light absorbance and its possible thermal effects that likely occur during PLFL. We present a simplified raytracing model for the calculation of the laser energy density distribution within the liquid jet and determine the particle size dependent absorption according to Mie theory^33^. Further details on our model and calculations of absorbed energies are to find in the supplementary information. The results are summarized in Fig. 8. For characteristic particle diameters (30, 50 and 200 nm) of the educt colloid, measured by intensity-weighted analytical disc centrifugation, we determine the absorbed laser energy in the different regions of the liquid jet during one laser pulse. Additionally, we calculate the same data for particles of 2 nm in diameter, because they represent the most dominant species by surface after the tenth PLFL passage. At this point, we have to mention that 13.8% of the jet cross-section is not irradiated with laser light due to refraction effects at the air-jet boundary. All following proportions are related to the whole jet cross-section. If we assume a total conversion of laser energy to thermal lattice energy and a homogeneous particle dispersion in the colloid, a full decomposition during one laser pulse is only possible for single particles or agglomerates of 200 nm in diameter, with 4.4%. Particles of the smaller regarded diameters cannot be fully decomposed. A complete particle melting is with 74.9% more probable for 200 nm particles. For particles of the other regarded diameters, entire melting is possible in 17.9% (50 nm), 14.1% (30 nm) and 10.7% (2 nm) of the cross-section.

We expect the different probabilities for material phase transformations and their dependence on the particle diameter to be the reason for the observed significant changes in the colloid extinction properties, particle size distribution and the chemical decomposition until the tenth PLFL passage. Furthermore, the occurrence of both mechanisms combined with the magnetically induced agglomeration of the particles will lead to multi-crystalline and multi-phase particles by fusion processes. Although, chemical decomposition due to thermal decomposition seems to be saturated after tenth passage, fusion processes are favoured towards renewed thermal decomposition and lead to the via HR-TEM observed network structures in products after the 25^th^ and 50^th^ passage (Supplementary Fig. S1), and probably to the further increase in catalytic activity due to an additional structural disorder. (We show an overview of the different products generated from the CoFe_2_O_4_ educt during PLFL in water in Supplementary Figure S8.)

## Conclusion

We present a dose-controlled method for enhancing the electrochemical catalytic activity for OER in alkaline media of a commercial CoFe_2_O_4_ nanoscale powder by PLFL in water. Drastic changes in the morphology, chemical composition and crystallinity take place during PLFL. We find ultra-small, amorphous particles and multi-crystalline spheres of diameters up to 200 nm as main products, as well as sheet-like structures as byproducts of PLFL. Whereas, the chemical composition of bigger spheres is occasionally shifted to a surplus of Co, we identify the sheet structures as Fe-based LDH. We further expect the composition of ultra-small, amorphous particles to be comparable to CoFe_2_O_4_ since superparamagnetism is strongly enhanced after tenth passage of PLFL. Our results indicate that thermal mechanisms, induced by laser light absorption of particulate matter, lead to particle decomposition and melting as well as fusion of agglomerated particles. As observed in the HR-TEM micrographs and shown by Mössbauer spectroscopy as well as the electrochemical investigation, structural disorder builds up during PLFL and improves the charge transport kinetics of the PLFL products compared to the CoFe_2_O_4_ nanoscale educt. Whereas, the light extinction properties and the chemical decomposition of the PLFL products reach saturation after the tenth passage, the electrochemical catalytic activity for OER is increasing until the 50^th^ passage, as well as morphology transformations due to particle fusion. The product after the tenth passage represents an optimum especially in terms of process efficiency. Along with the mentioned material properties, also the overpotential for the investigated OER enters a saturation at 0.345 V.

The comparability of the performance of our catalysts to others published in literature is limited due to its complex structure and differences in the conditions of the electrochemical measurements. However, we choose catalysts of mixed oxides of Co and Fe deposited on glassy carbon and tested in alkaline media as benchmarks. Compared to McCrory *et al*. who synthesized a CoFeO_x_ catalyst by electrodeposition and achieved an overpotential of 0.37 V at 10 mA cm^−2^ we are able to reduce the overpotential by 6.8% and 13.5% for the product after the tenth and 50^th^ passage, respectively^11^. They also tested two CoO_x_ catalysts at the same conditions and measured overpotentials of 0.39 and 0.42 V. With respect to our lower overpotentials of 0.345 V (tenth passage) and 0.32 V (50^th^ passage) a synergy effect of CoFe_2_O_4_ and CoO can be assumed for our catalysts. Indra *et al*. compared amorphous and crystalline CoFe_2_O_4_ catalysts in 0.1 M KOH and obtained overpotentials of 0.49 and 0.56 V, respectively^43^. The better performance of amorphous CoFe_2_O_4_ is interesting since the fraction of ultra-small particles in our experiments is amorphous as well.

While the complexity of the product of PLFL causes the superior electrochemical properties, it also complicates a clear assignment of their origin. The presence of LDH additionally affects the activity for OER positively, especially for the products after the 25^th^ and 50^th^ passage of PLFL. Hence, the role of the CoFe_2_O_4_-CoO-coexistence and the superparamagnetic particle fraction in the catalytic activity needs to be further clarified. A separation of both species, via centrifugation for instance, is not accessible due to magnetic polarization effects. Thus, we need to clearly address the synthesis of the single species within the PLFL process. As our model suggests, this should be achievable by a more homogeneous distribution of energy density within the liquid jet, which probably enables a control of the occurring thermal mechanisms by the applied laser power. Furthermore, we need to reduce the number of laser pulses irradiating a defined volume fraction of the colloid in one passage to one to avoid multiple transition processes during one PLFL passage.

## Methods

### Pulsed laser fragmentation in liquid (PLFL)

PLFL was performed with a pulsed Nd:YAG laser (Atlantic, Ekspla, Vilnius, Litauen) with a pulse duration of 10 ps and, operated in the second harmonic, a wavelength of 532 nm with a measured power of 7.2 W. The applied repetition rate was 100 kHz, resulting in a pulse energy of 72 µJ. The setup consisted of the described picosecond laser, a cylindrical lens, a passage reactor (Fig. 8a), a beaker and a power meter. The raw laser beam of 2 mm in diameter was focused at its vertical axis to a diameter of 44 µm, calculated by Supplementary Equ. S5, by the cylindrical lens. An average fluence of 104 mJ cm^−2^ is reached. From a capillarity with a measured diameter of 1.3 mm at the bottom of the passage reactor a CoFe_2_O_4_ powder (Sigma Aldrich, St. Louis, USA), dispersed in water, flew centred through the focus position of the laser beam. The irradiated colloid (0.05 wt.-% of CoFe_2_O_4_ in H_2_O) was collected in a beaker and afterwards refilled into the reactor. This procedure is called a passage and is repeated for 50 times maximum in our experiments. Power loss measurements were performed by utilization of a thermopile sensor (PowerMax PM30, Coherent, Santa Clara, USA).

### General sample preparation for analytics

For all analytical measurements described here, when not mentioned in a different way, the colloid was dried after PLFL in a crystallization tray at 70 °C for 12 h in air. For the separation of non-particle species from some products after the tenth passage of PLFL, a Optima MAX-XP (Beckman Coulter, Brea, USA) ultracentrifuge was used.

### UV-vis extinction spectroscopy

Extinction spectroscopy was applied before drying the colloids to powders. Colloid samples (700 µL) were filled into a quartz glass micro cuvette with 10 mm beam way. All extinction spectra were recorded at an extinction calibrated Evolution 201 (Thermo Fisher Scientific, Waltham, USA) spectrometer in the range of 190 to 1,100 nm.

### Powder X-ray diffraction (PXRD) analysis

PXRD patterns were obtained using a D8 ADVANCE (Bruker, Billerica, USA) powder diffractometer with Cu Kα radiation (λ: 1.5418 Å, 40 kV and 40 mA) using a silicon single crystal as sample holder to minimize scattering. For better homogenization, the dried powder samples were re-dispersed in ethanol on the silicon surface and then investigated in the range from 10° to 90° 2 θ with a step size of 0.01° 2 θ with a counting time of 0.6 s. Rietveld refinement was performed with the program package TOPAS 5.0 (Bruker) to determine the lattice parameters. The background was modelled using Chebyshev polynomials.

### High-resolution transmission electron microscopy (HR-TEM)

HR-TEM was performed at a JEM-2200FS (JEOL, Akishima, Japan), as well as the energy dispersive X-ray spectroscopy (EDX). Preparation of TEM grids was done by ten times diluting the colloid with water and directly dropping it on a carbon-coated copper grid and in some cases on a holey carbon grid because of the low contrast between Fe, Co and Cu.

### Mössbauer spectroscopy

Mössbauer spectra were recorded in transmission geometry using a constant acceleration Mössbauer driving unit with a ^57^Co source embedded in a Rh matrix. The spectrometer was calibrated with a α-Fe foil reference sample at room temperature. Low temperature and in-field spectra were measured in a liquid helium bath cryostat with a superconducting magnet in split-coil geometry, providing magnetic fields of up to 5 T along the γ-ray propagation direction.

### X-ray photoelectron spectroscopy (XPS)

XPS measurements were performed at a VersaProbe II (Ulvac-Phi, Chanhassen, USA). Droplets of the different powder samples, redispersed in water, were put on cleaned copper foil. Both Al Kα and Mg Kα light was used to investigate the powder samples. No qualitative difference in the Co and Fe spectra were found for the two wavelengths.

### Electrochemical analysis

All electrochemical measurements were performed in a conventional three-electrode cell using an Autolab potentiostat/galvanostat (PGSTAT12, Eco Chemie, Utrecht, The Netherlands) coupled to a rotating disk electrode rotator (Metrohm, Herisau, Switzerland). Disc shaped glassy carbon of 0.126 cm^2^ geometric area, modified with the catalysts was used as the working electrode, Ag/AgCl/3 M KCl as the reference electrode and a Pt mesh as the counter electrode. The measured potentials were converted to the reversible hydrogen electrode (RHE) scale using the following equation *E(RHE)* = *E(Ag/AgCl)* + 0.210 V + 0.059 x pH V. The pH value was determined (using a pH meter) and was 14 for 1 M KOH. Prior to the experiments, the glassy carbon electrode was polished with a polishing cloth using different alumina pastes (3.0–0.05 μm) to obtain a mirror-like surface, followed by ultrasonic cleaning in water. For electrochemical measurements, the catalyst ink was prepared by dispersing the catalyst suspension (5.0 mg mL^−1^) in an ethanol-water mixture (1:1) and ultrasonicated for 30 min. The catalyst ink (5.0 μL) was drop coated onto the polished glassy carbon electrode and dried in air at room temperature. Before performing the OER measurements, modified electrodes were subjected to continuous potential cycling in the potential window of 0.1 V to 1.1 V vs. RHE until reproducible voltammograms were obtained. Electrochemical impedance spectroscopy was then recorded in the frequency range from 50 kHz to 1 Hz at the corresponding open circuit potential of the electrode, using an AC perturbation of 10 mV. The resistance of the solution was determined from the resulting Nyquist plot and the later used for ohmic drop correction according to the relation, *E*_*c*_ = *E*_*m*_ – i *R*_*s*_, where *E*_*c*_ is the corrected potential and *E*_*m*_ is the applied potential. All reported current densities were calculated using the geometric surface area of the electrode.

### Data Availability

All data generated and analyzed, except some PXRD data used for phase assignment (see below), during this study are included in this published article and its Supplementary Information file at least graphically in diagrams. The tabulated datasets are available from the corresponding author on request. PDF numbers of different crystalline phases used for x-ray diffraction pattern analysis are: 00-003-0864 [CoFe_2_O_4_], 00-001-1227 [CoO] and 00-046-0098 [Fe_6_(OH)_12_CO_3_]. The data sheet of the used CoFe_2_O_4_ educt powder is available online: http://www.sigmaaldrich.com/Graphics/COfAInfo/SigmaSAPQM/SPEC/77/773352/773352-BULK_______ALDRICH__.pdf (availability checked at 05.05.2017). The company’s catalogue number of the product is 7752252.

## Electronic supplementary material

Supplementary Information
