# Supplementary material for: Adjusting the catalytic properties of cobalt ferrite nanoparticles by pulsed laser fragmentation in water with defined energy dose
Source: Sci Rep. 2017 Oct 13;7:13161. doi: 10.1038/s41598-017-13333-z (PMC5640596; doi:10.1038/s41598-017-13333-z)
Supplement: Supplementary file 1 — Supplementary Information [file 41598_2017_13333_MOESM1_ESM.pdf]

Adjusting the catalytic properties of cobalt ferrite nanoparticles by pulsed laser fragmentation in water with defined energy dose

Friedrich Waag, Bilal Gökce*, Chakrapani Kalapu, Georg Bendt, Soma Salamon, Joachim Landers, Ulrich Hagemann, Markus Heidelmann, Stephan Schulz, Heiko Wende, Nils Hartmann, Malte Behrens*, Stephan Barcikowski

All additional data shown here only complements that of the main manuscript. We provide a detailed explanation of the applied modelling of the laser beam pulse energy density at the end of this supporting information. The explanation also contains the comparison of by single particles of different sizes absorbed energy according to Mie theory to energy thresholds for thermal phase transitions, melting and decomposition, in particular.

High-resolution transmission electron microscopy (HR-TEM)

We want to present additional HR-TEM data here. Supplementary Figure S1 shows representative micrographs and the particle size histograms for the educt and products after different passages of PLFL. For all histograms, more than 1,000 particles are measured. Layered double hydroxide (LDH) sheets and molten network structures, we do not take into account for these size measurements. Very small particles of around 2 nm in diameter show just a slight contrast to the support grid and are only present in conglomerates, for this reason a clear particle border was difficult to identify and we need to choose a broad size fraction interval of 4 nm to consider the error of measurement. Furthermore, we need to assume, that smaller particles attach on top or underneath bigger particles. Thus, the fraction of small particles is probably underestimated. Additional problems in determination of the particle diameters from TEM micrographs are caused by the high agglomeration of particles especially at lower PLFL passages. The agglomeration is probably enhanced due to the ferrimagnetism of $CoFe_2O_4$. At higher passage numbers, the agglomeration of bigger particles on the TEM grid is reduced due to steric shielding caused by the high amount of superparamagnetic particles.

The TEM micrographs illustrate how the morphology of particles changes within the PLFL process (Supplementary Fig. S1). Whereas the educt particles have a crystal-like shape, the product particles are spherical. In the product after the tenth passage, we cannot find any crystal-like particles, but we can find them in that after the fifth passage. Conclusively, a repetition of somewhat between five and ten passages is needed to induce a transformation of each educt to product particles. We expect that this is due to a not complete illumination of the colloid which is caused by optical refraction of the laser light at the border of the liquid jet. However, the morphology change does not stop after the tenth PLFL passage. The amount of very small particles is high in the product after the tenth passage, and later on at higher passage numbers, those particles seem to melt together and fuse with bigger particles. We can nicely see the result of this effect in the micrograph of the product after the 50th passage. In addition, sheet structures are visible in micrographs of the product after the 25th passage (near the right border) and after the 50th passage (top left corner). We will regard their formation in detail later in this supporting information.

From the histograms in Supplementary Fig. S1, we can clearly see that the surface contribution of very small particles increases drastically up to the product after the tenth passage. The broad size distribution of the educt tightens, but we can still find some spheres with more than 100 nm in diameter in all products, expect in that after the 50th passages, but this is possibly due to the method of measuring sizes by a few TEM micrographs per sample. The formation of the spheres we can relate to melting processes of agglomerates, which explain the perfect spherical shape.

We show an additional elemental mapping by energy dispersive X-ray spectroscopy (EDX) of a large section of a TEM grid coated with the product after the tenth passage of PLF in Supplementary Fig. S2. It contains hundreds of bigger spheres that exhibit a homogeneous distribution of Co, Fe and O. By investigation of the ratio of Co to Fe of single particles, we find occasional deviations in stoichiometry of around 1:1 compared to the expected ratio of

1:2. This affects approximately one of 100 investigated particles. Considering the high amount of crystalline CoO (around 60 vol.-%) in that sample, it is highly unlikely to have no other Fe phase present in the particles. We will discuss the role of Fe in the decomposition of $CoFe_2O_4$ in detail in the sections of PXRD and the formation of LDH.

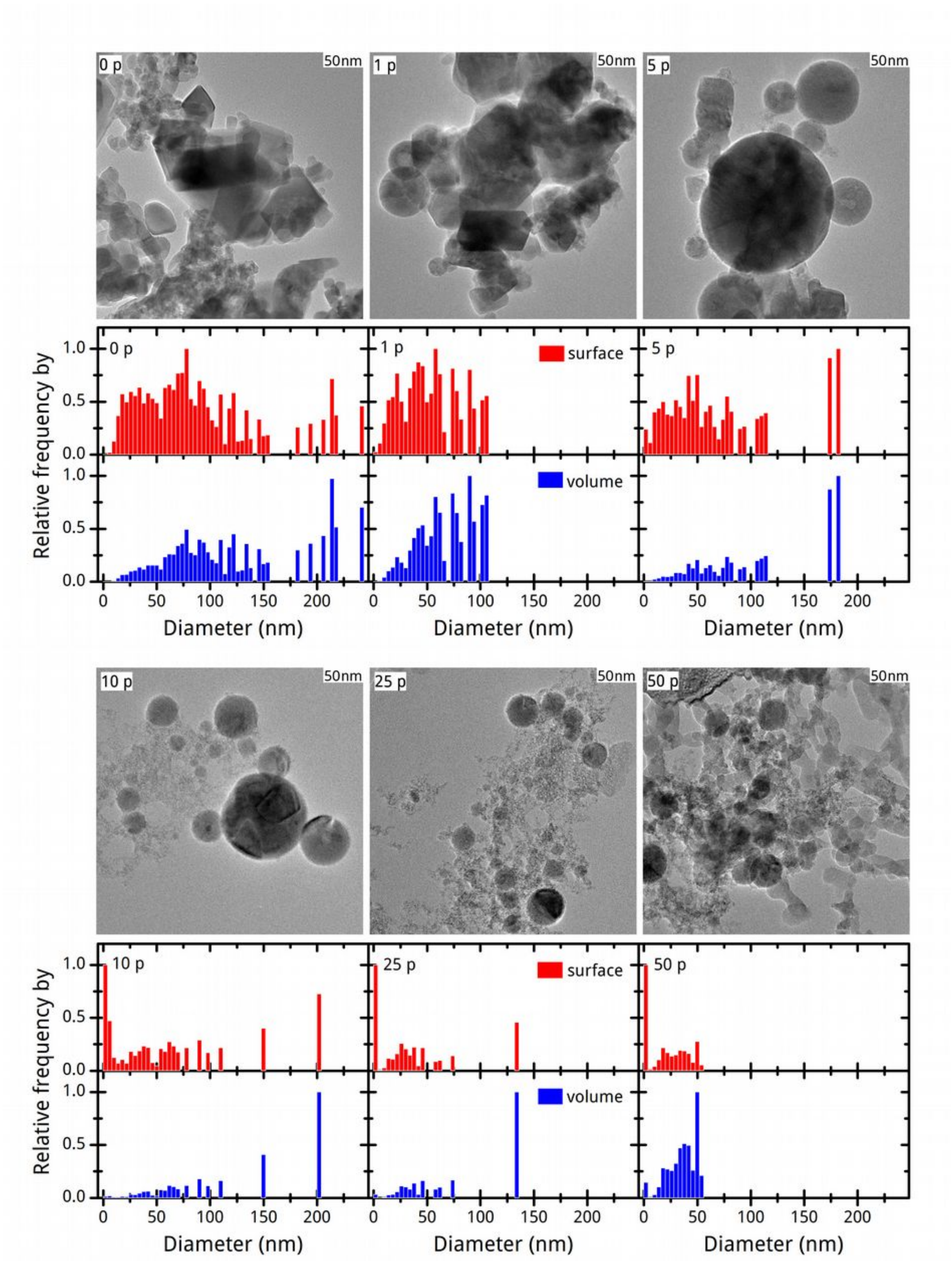

Supplementary Figure S1: HR-TEM micrographs and particle size histograms, volume and surface weighted, for the educt and products after first, fifth, tenth, 25th and 50th passage. Molten network structures as well as LDH sheets (mainly in products after 25th and 50th passage) are not taken into account for histograms.

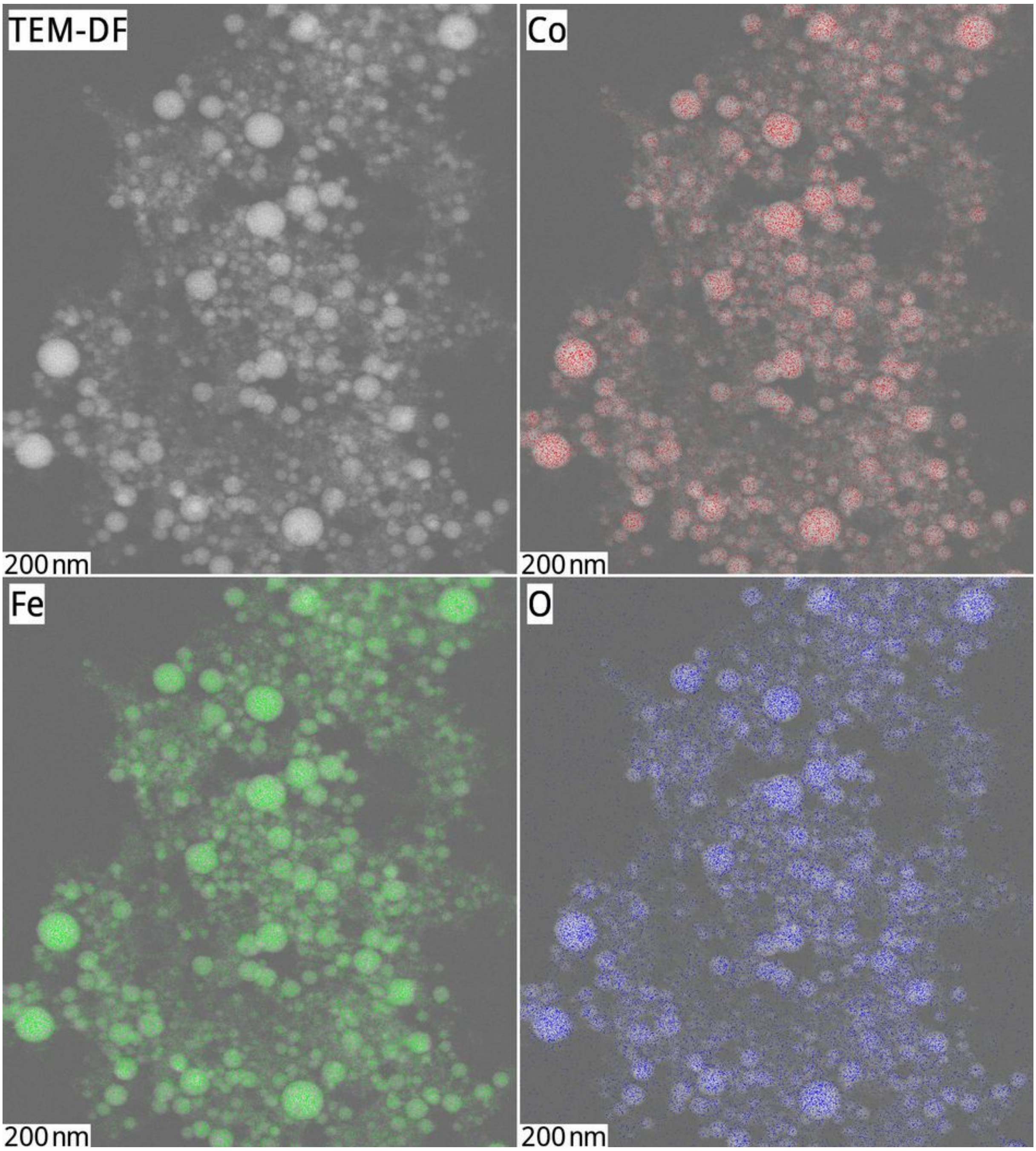

Supplementary Figure S2: Dark field (DF) micrograph via TEM of the product after the tenth passage and EDX-mappings of Co, Fe and O.

Surface area by BET method

To determine the effect of the strong morphology change observed by the TEM analysis on the potentially available surface for catalytic interactions of the samples, we measure the specific surface area by the established method of Brunauer, Emmett and Teller (BET) for nitrogen adsorption[nS1]. Beside the educt, we choose two products of PLFL, after the first and

tenth passage, for the BET analysis. The results are 32, 30 and 42 m^2/g, respectively. The BET surface areas do not show a clear dependence on the number of applied PLFL passages. At least for the product after the first passage of PLFL, we would have expected a BET surface area slightly higher than that of the educt according to the particle size distribution from the TEM analysis. A possible reason for the obtained result could be agglomeration of the particles during drying, which should be much stronger for the laser-treated particles due to the decomposition of ligands on the educt particle surfaces. The latter increase in the BET surface area for the sample after the tenth passage could be explained by the strong size reduction caused by PLFL up to that passage. However, the BET surface area does not correlate to the change of the electrochemical properties of the samples in this case.

Powder X-ray diffraction (PXRD)

We investigate the transition of crystalline phases during PLFL processing by PXRD, as described in the main text and shown in Fig. 4. A partly transition of $CoFe_2O_4$ to CoO directly in the first passage is obvious. We calculate the lattice strain for the CoO-related refraction peaks to check whether they could be also connected to FeO. If we assume the refraction to originate from a CoO lattice, the strain of the lattice parameter *a* would be -0.28 % compared to a bulk CoO[S2]. In comparison to a FeO bulk, we would get a strain of -1.64 %[S3]. Both strains are compressions, whereas a CoO lattice is more likely due to the by six times lower compression. However, both relative strains a quite low and an exclusion of FeO or $Fe_xCo_{1-x}O$ as crystalline phase only by PXRD data is difficult. In addition, the lattice parameter *a* of the $CoFe_2O_4$ phase has a strain of 0.08 %[S4]. (We averaged the strain for all phases over all clearly assignable refractions and of all products without seeing a trend to the PLFL passage number.) The CoO phase reaches a saturated volume amount (relative to $CoFe_2O_4$ + CoO) of 0.61 after the tenth passage.

In Supplementary Fig. S3a, we present the raw diffractograms of proceeded PXRD measurements, not background corrected and measured without monochromator. The background contains information about fluorescent species that are excited by the used Cu Kα radiation of the diffractometer, which is the case for Co and Fe, and x-ray diffraction at non-crystalline species[S5]. Thus, the high backgrounds of all our measurements are not surprising because of the fluorescence active Fe and Co, but interestingly the slope of the background changes significantly already after the first passage of PLFL and changes back after the fifth passage. In products after higher passage numbers, the slope is not varying that much again. The sample amounts and measuring time are comparable for all measurements. This is not turning the background into a source of quantitative information, but a by the half higher background intensity at high 2 θ values in the product after the fifth passage compared to the educt and product after the first passage is not negligible. Thus, we have probably two different varying background contributions, one affecting the intensity at small and one that of high 2 θ values. For further investigation, we centrifuge a product after the tenth PLFL passage and measure the dried sediment (Supplementary Fig. S3b). (We chose the product after the tenth passage because of the reached saturation in investigated extinction data (Fig. 1b/c).) The background falls down drastically in the range of small 2 θ values but stays unchanged at high ones. Conclusively, species affecting the intensity at small angles are removed during centrifugation and those affecting the intensity at higher angles remain. The centrifugation parameters are suitable for selectively removing the low density LDH and ion complexes from the sample. The success is confirmed by UV-vis extinction investigation (Supplementary Fig. S3b) and TEM (Supplementary Fig. S5). The supernatant is not containing any particulate matter but shows a sharp absorption shoulder in the UV range indicating the presence of Fe and/or Co species. Conclusively, we assign the background contribution at small angles to LDH precursors. At higher angles, we expect the small particle fraction, which is amorphous, to mainly contribute to the background. Otherwise, we also

investigate a product after the tenth passage of PLFL in acetone by PXRD (Supplementary Fig. S3b). The diffraction pattern looks similar to the one of the sample fragmented in water. A formation of LDH and its precursors in acetone is unlikely since the solubility of Fe and Co is strongly limited compared to water. However, the amount of CoO in the sample is comparable to the sample produced in water. This result is controversial and requires further studies for clarification.

We further investigate the effect of the applied PLFL process on powders of CoO and Fe_2O_3 in which Co and Fe have the same valences as in $CoFe_2O_4$. The PXRD results are presented in Supplementary Fig. S3c/d. Interestingly, CoO completely transforms into Co_3O_4. This spinel shows the same refraction pattern like Fe_3O_4 and $CoFe_2O_4$ but due to its smaller lattice constant shifted to higher 2 θ values. If we relate the observed PXRD pattern of the $CoFe_2O_4$ products to Co_3O_4, there would be strain of the lattice constant *a* of 3.86 % compared to bulk Co_3O_4[S6]. Since the strain of *a* for $CoFe_2O_4$ or Fe_3O_4 would be only 0.08 %, we expect them to originate the observed pattern. Furthermore, Fe_2O_3 also completely transforms to the spinel. Here we need to mention that the educt (purchased at Sigma Aldrich) already contains a high amount of Fe_3O_4. Fe_3O_4 is not distinguishable from $CoFe_2O_4$ by analyzing its crystal lattice, though a presence in our $CoFe_2O_4$ samples is not detectable by PXRD. The behavior of CoO in presence of $CoFe_2O_4$, and possibly Fe_3O_4, during PLFL is different from that in the single phase processing: Co is partly oxidized in the supplemented experiment and seems to be unchanged in oxidation state in PLFL of $CoFe_2O_4$. We assume the less strained crystalline phases, $CoFe_2O_4$ and/or Fe_3O_4 and CoO, to primarily contribute to the refraction patterns of our PLFL products. Furthermore, the results of XPS and Mössbauer spectroscopy do not indicate the existence of other phases than observed by PXRD. However, it is interesting that during PLFL of single-phase powders only the spinel phase formats.

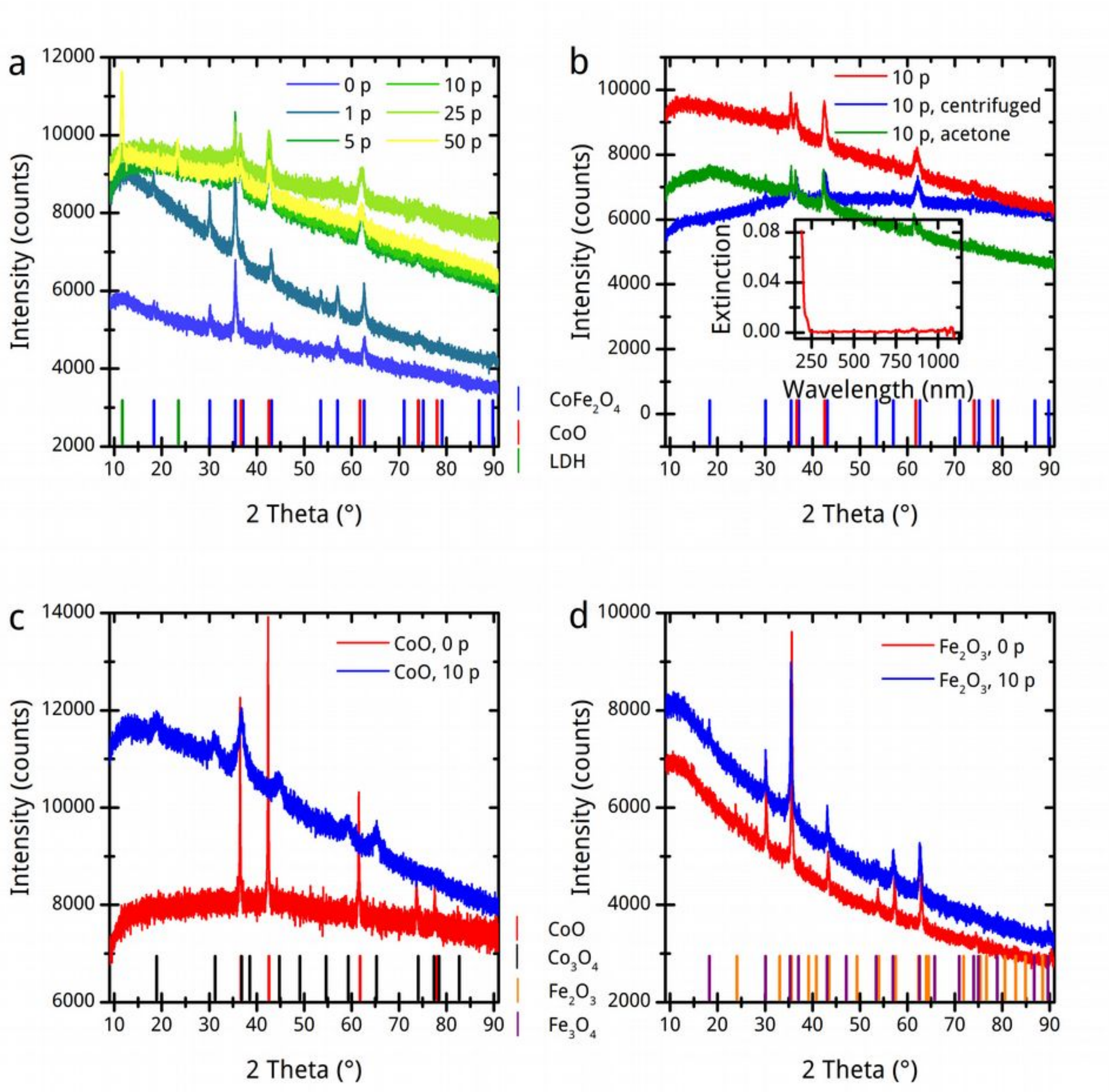

Supplementary Figure S3: Raw PXRD patterns of $CoFe_2O_4$ PLFL samples after different passages of PLFL (a), comparison of a product after tenth passage before and after centrifugation (dried sediment) as well as of a product after tenth passage of PLFL in acetone (b), as well as of CoO (c) and Fe_2O_3 (d) before and after tenth passage, respectively. The inlet of (b) shows the UV-vis extinction spectra of the supernatant of the centrifuged sample.

Formation of layered double hydroxide (LDH)

As mentioned in the main manuscript and before in this supporting information, we want to discuss the formation of observed LDH species more in detail here. Supplementary Figure S2 shows TEM micrographs of different LDH sheets found in products after 25th and 50th passage of PLFL. The sheets clearly grow with number of passages and have dimensions of more than

200 nm in the product after the 50th passage. The contrast is also strengthening. Thus, we can assume that the number of layers is also increasing. We can find single LDH sheets at first in the product after the tenth passage, and it is possible to remove them from the colloid by centrifugation, as already mentioned. Of one LDH sheet of the supernatant, we proceed an EDX element mapping, shown in Supplementary Fig. S3. Furthermore, this figure shows the atomic lattice of one LDH sheet we found in the product after the 25th passage of PLFL. This LDH sheet mainly builds up on Fe and has only a weak cobalt Co signal in the range of the noise. The lattice is clearly hexagonal with the lattice parameter $a = (2.9 \pm 0.1)$ Å. This is in good agreement with common Fe-based LDH compounds[S7]. A partial reduction of Fe, needed for LDH formation, can be induced by the laser irradiation as recently shown by Ishikawa et al.[S8].

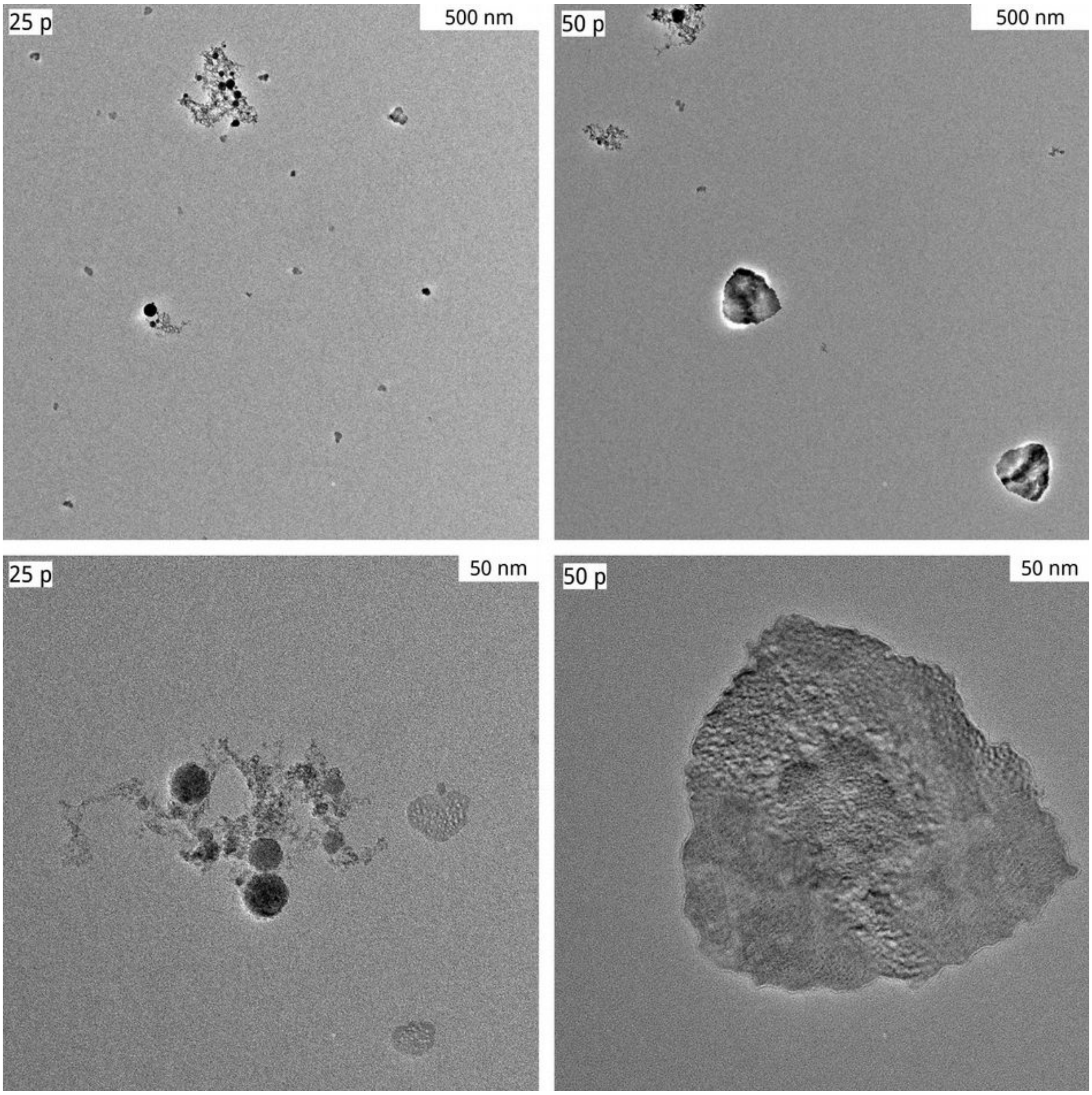

Supplementary Figure S4: TEM micrographs of products after 25th (left) and 50th (right) passage containing representative LDH sheets.

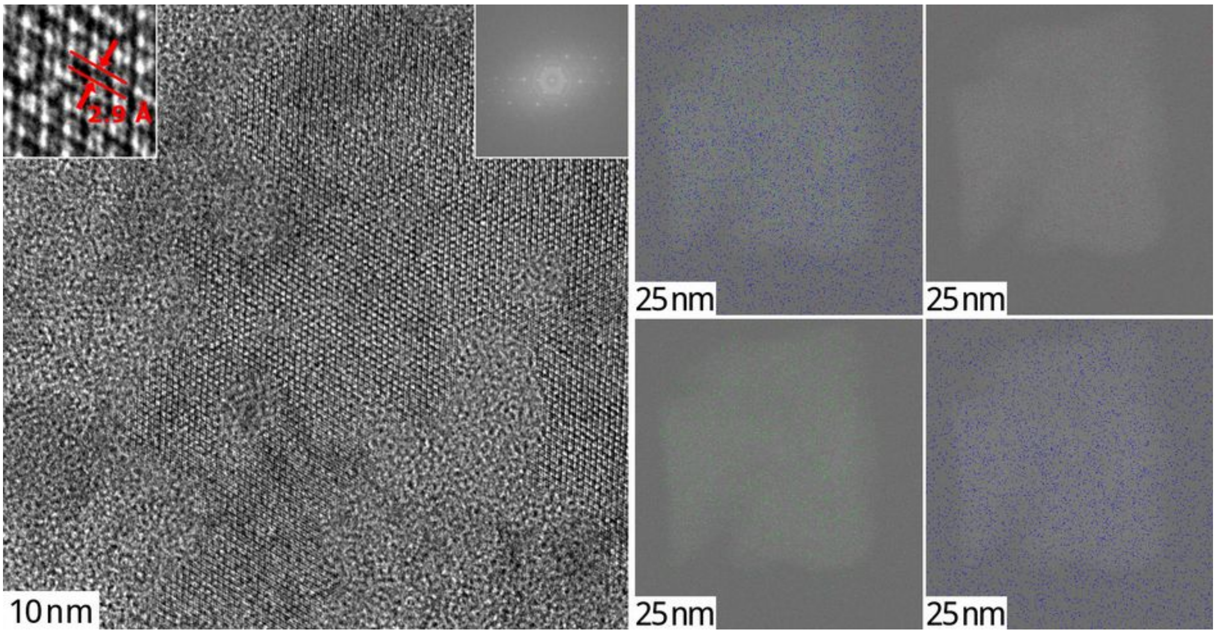

Supplementary Figure S5: HR-TEM micrograph of a LDH sheet found in the product after 25th passage (left) and EDX element mapping of an LDH sheet found in the supernatant of a centrifuged product after the tenth passage (right). The inlays of the micrograph on the left show the real and reciprocal lattice. The lattice is clearly of hexagonal type with a lattice parameter $a = b = (2.9 \pm 0.1)$ Å. Mappings of the kα emission of Co, Fe and O on the right exhibit a homogeneous distribution of Fe in the LDH sheet while the signal of Co is not significant and that of O already quite high in the background.

The decomposition of $CoFe_2O_4$ to CoO releases Fe that converts at least partly into the observed LDH phase. We expect that Fe species solve into the surrounding water during the decomposition and are present as oxyhydroxide species at the pH of 8.6, we measure for the colloids. Laser energy that couples into the liquid triggers the formation and growth of the LDH sheets. Synthesis routes for LDH are manifold and can be proceeded at different pH values starting at various precursors[S9]. Hunter et al. published a laser synthesis of LDH comparable to our expected formation mechanism[S10]. They irradiated an alkaline metal salt solution containing micron scaled grains of another metal with a laser to synthesize bimetallic LDH. In the product after the 50th passage, the LDH phase shows up in the recorded PXRD diffractogram (Fig. 4). The two main refraction peaks are related to [003] and [006] plane distances[S11]. Based on the refraction angles, we calculate the interplane distances and subsequently the lattice parameter c as 22.87 Å. It is best fitting to a Fe-based LDH with incorporated carbonate anions[S7]. In products after lower investigated passage numbers than

the 50th, the volume amount of LDH is apparently lower than the determination limit of the diffractometer.

Electrochemical analysis

Beside the correlation of electrochemical and material properties presented in the manuscript, we also proceed additional experiments to obtain further information about the mechanism of the activity improvement. We already presented those experiments shortly in the XRD part of the supplementary information. The first one is the centrifugation of a product after the tenth passage of PLFL to remove LDH from the sample, the second one is the PLFL of a CoO dispersion for also ten passages, and the third experiment is the PLFL of the $CoFe_2O_4$ educt in acetone instead of water for ten passages to prevent the formation of LDH. We need to note here that all these experiments have their limitations in comparability to the main experiments. The centrifugation leads to agglomeration and aggregation of the nanoparticles. Because of the quite low density of $CoFe_2O_4$ and the very small particle size, high g-forces need to be applied. For the other two experiments, we need to mention that changing the to be fragmented material or the liquid environment will strongly affect the chemical processes during laser irradiation. In the supplementary XRD section, for example, we show that the PLFL of CoO in water leads to Co_3O_4. Thus, the here presented results are limited in their comparability to the results of the PLFL of $CoFe_2O_4$ in water.

Supplementary Fig. S6a shows the linear sweep voltammograms of the centrifuged $CoFe_2O_4$ sample after tenth passage of PLFL and $CoFe_2O_4$ sample after the tenth passage of PLFL in acetone in comparison to the $CoFe_2O_4$ educt and the product after the tenth passage of PLFL in water. Interestingly, the centrifuged and the in acetone produced sample both show a nearly identical graph by observing a slightly better performance for the centrifuged sample. In comparison to the $CoFe_2O_4$ educt and the product after the tenth passage of PLFL in water,

their overpotentials at 10 mA cm^{-2} are located nearly in the middle. This result could indicate that there is a bigger contribution of LDH to the activity of the product after the tenth passage as indicated by the results shown in the manuscript.

Supplementary Fig. S6b shows the linear sweep voltammograms of a CoO educt and product after the tenth passage of PLFL in water in comparison to the $CoFe_2O_4$ educt and the product after the tenth passage of PLFL in water. The CoO educt already shows a good performance for low current densities. But the performance decreases drastically for higher currents densities. The overall graph looks comparable to that of the centrifuged $CoFe_2O_4$ sample in Supplementary Fig. S6a. The PLFL increases the slope to a comparable value to the $CoFe_2O_4$ sample after the tenth passage of PLFL. Since the PLFL of CoO in water results in Co_3O_4, as shown by PXRD, the results are not helpful in clarifying the mechanism of activity improvement of the products of PLFL of $CoFe_2O_4$ in water.

Conclusively, these additional experiments only underline the uniqueness of the product of the PLFL of $CoFe_2O_4$ in water and that there are different contributions of that product to the activity improvement. We also find here a slight hint for a higher contribution of the formed LDH species, but this is only one possible interpretation of the obtained results.

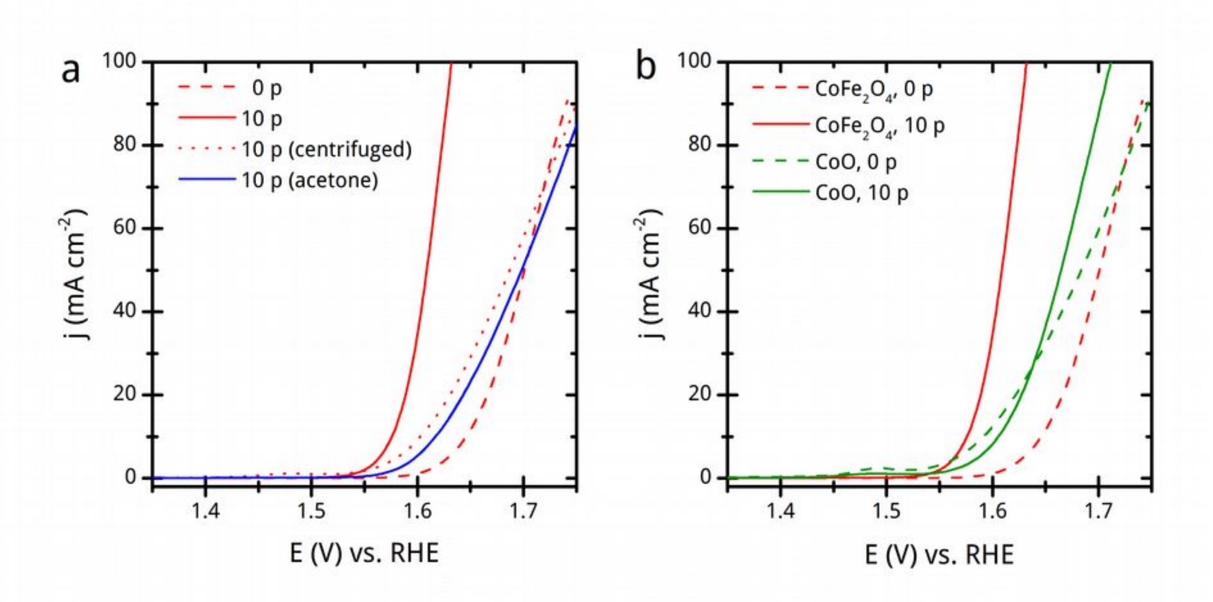

Supplementary Figure S6: Linear sweep voltammograms of a centrifuged $CoFe_2O_4$ sample after the tenth passage of PLFL and $CoFe_2O_4$ sample after the tenth passage of PLFL in acetone in comparison to the $CoFe_2O_4$ educt and the product after the tenth passage of PLFL in water (a), of a CoO educt and product after the tenth passage of PLFL in water in comparison to the $CoFe_2O_4$ educt and the product after the tenth passage of PLFL in water (b).

Pulsed laser fragmentation in liquid (PLFL)

At first, we want to present the important parameters of the used laser system. We use an Ekspla Atlantic 1064/532 for all experiments. The laser's beam quality k is 0.77, the pulse length t_{lp} is given as 10 ps, the applied repetition rate R is 100 kHz and the wavelength λ of the used second harmonic 532 nm. In addition, the laser raw beam with a diameter d_b at $1/e^2$ of 2 mm is focused at its vertical axis by use of a cylindrical lens with a focal length f of 100 mm. All laser parameters, given in this section, are data of the distributors and have not been measured by us. We measure the laser power with a thermopile sensor as 7.2 W.

The process of pulsed laser fragmentation in a thin liquid jet has some advantages compared to other methods published by the community. One advantage is the possibility of calculating the average number of interactions of a particle or volume unit with the laser beam in one passage of PLFL and based on this the specific energy intake of the particulate matter. In a first step, we calculate the outflow velocity v_o of the liquid jet on the colloid volume V_c, the circular capillarity cross-section area A_j, that is expected to be the cross-section area of the

liquid jet also, and the outflow time to of the specific colloid volume (Equation S1). Furthermore, the number of pulses N_p illuminating a specific outflowing volume fraction during one PLFL passage is determined by the height of the laser spot h_s at the liquid jet on its vertical cross-section and R (Equation S2). Caused by the waist of the jet, h_s is varying from a value of 48.4 µm (Equation S3) to the focus diameter d_f in dependence to the distance to the focus on the axis of irradiation x_i. For the calculation of h_s the Rayleigh length l_R is needed (Equation S4). We perform the calculation of d_f by using Equation S5 and it results in 44.0 µm in our case. Further focusing due to refraction at the jet border is ignored here, because of the small incident angle of 0.4 °. We approximate the value for h_s to 46 µm in further calculations. The results for v_c and N_p are 500 mm s^{-1} and 9.2, respectively.

$$v_o = V_c / A_j \tag{S1}$$

$$N_p = R \cdot h_s / v_o \tag{S2}$$

$$h_s = d_f \cdot \sqrt{1 + \left(x_i / l_R\right)^2} \tag{S3}$$

$$l_R = \pi \cdot \left(d_f / 2\right)^2 / \lambda \tag{S4}$$

$$d_f = 4 \cdot \lambda \cdot f / \left(\pi \cdot d_b \cdot k\right) \tag{S5}$$

For the calculation of the specific energy intake of each particle during one laser pulse, we determine the irradiated colloid volume V_{ic} first. We simplify V_{ic} to a cylinder with A_j as base and h_s as height. Actually, V_{ic} should be regarded as the intersection of the circular jet and the elliptic beam cylinder volume. However, the beam ellipsis is just slightly bended in the area of beam penetration of the liquid jet because of the big differing lengths of both half axes of the beam (23 µm and 1000 µm) and therefore regarded as a straight line. The volume concentration c_V of $CoFe_2O_4$ in water is 0.0094 vol.-% and the measured power difference between an irradiated water jet and the colloid jet is 1.0, 0.8 and 0.6 W for first, second and all other performed passages of PLFL, respectively. A decrease of the power can be triggered by a partly decomposition of solid particle matter to solved ions and/or a less strong shielding

effect caused by the decreasing average particle diameter d_P. At given R and V_c of 6.1 × 107 µm³, the specific energy dose of the colloid is 164, 131 and 98 mJ cm^{-3} per laser pulse of first, second and each following passage, respectively. After extrapolation to whole passages, by multiplication with 9.2, we receive 1.51, 1.21 and 0.90 J cm^{-3}. These values relate to the total colloid volume. A relation to the total particle volume is easily done by multiplying the colloid volume with non-percentage c_V before dividing the energy amount, because the measured power is already corrected in terms of water absorption due to the differential measuring procedure. Related to the total particle volume, we receive 16.06, 12.87 and 9.57 kJ cm^{-3} for first, second and each following passage of PLFL or 1.74, 1.40 and 1.04 kJ cm^{-3} for a single laser pulse during first, second and each following passage.

The energy intake is related to all solid matter in an irradiated colloid volume. To distinguish the absorbed energy of single particles in dependence to their size and position in the liquid jet during the irradiation, we model the energy density distribution within the irradiated jet volume. Our model is based on a raytracing approach and considers one single laser pulse without time resolving. Firstly, we make some assumptions and simplifications to reduce the effort of calculation. The resolution of the model is set to one ray per 5 µm in the horizontal plane. The resolution of the horizontal direction of irradiation is also set to 5 µm and the vertical axis, in liquid jet flow direction, is not resolved. We only show the 5 µm thick center plane of the Gaussian beam in vertical direction. We expect the laser beam profile to be perfectly Gaussian-like in TEM00 mode and the liquid jet to be a perfect cylinder with static and smooth water-air boundary. A distribution of laser's wavelength is ignored as well as light reflection at the inner boundary of the jet. Furthermore, we apply all described experimental settings to the model.

Due to the Gaussian-like distribution, each ray carries another energy. Each ray interacts with the boundary of the liquid jet and the surrounding air, according to Fresnel equation and its form in special case of equated phase factors known as Snell's law. We assume commonly

known parameters of air and water here. Within the liquid jet, the single rays spread linear and their energy decreases due to extinction by the colloid according to Beer's law. We extract the colloid's extinction coefficient from UV-vis extinction data at 532 nm. The extinction coefficient is not constant for all passages as shown in Supplementary Fig. S7a. This is of course due to changes in morphology and chemical composition of the colloid. We choose a coefficient of 2.4×10^4 cm^{-1} like it is a god average for first passages of PLFL. The contribution of water to the coefficient is with 3.5×10^{-4} cm^{-1} negligible low[S12].

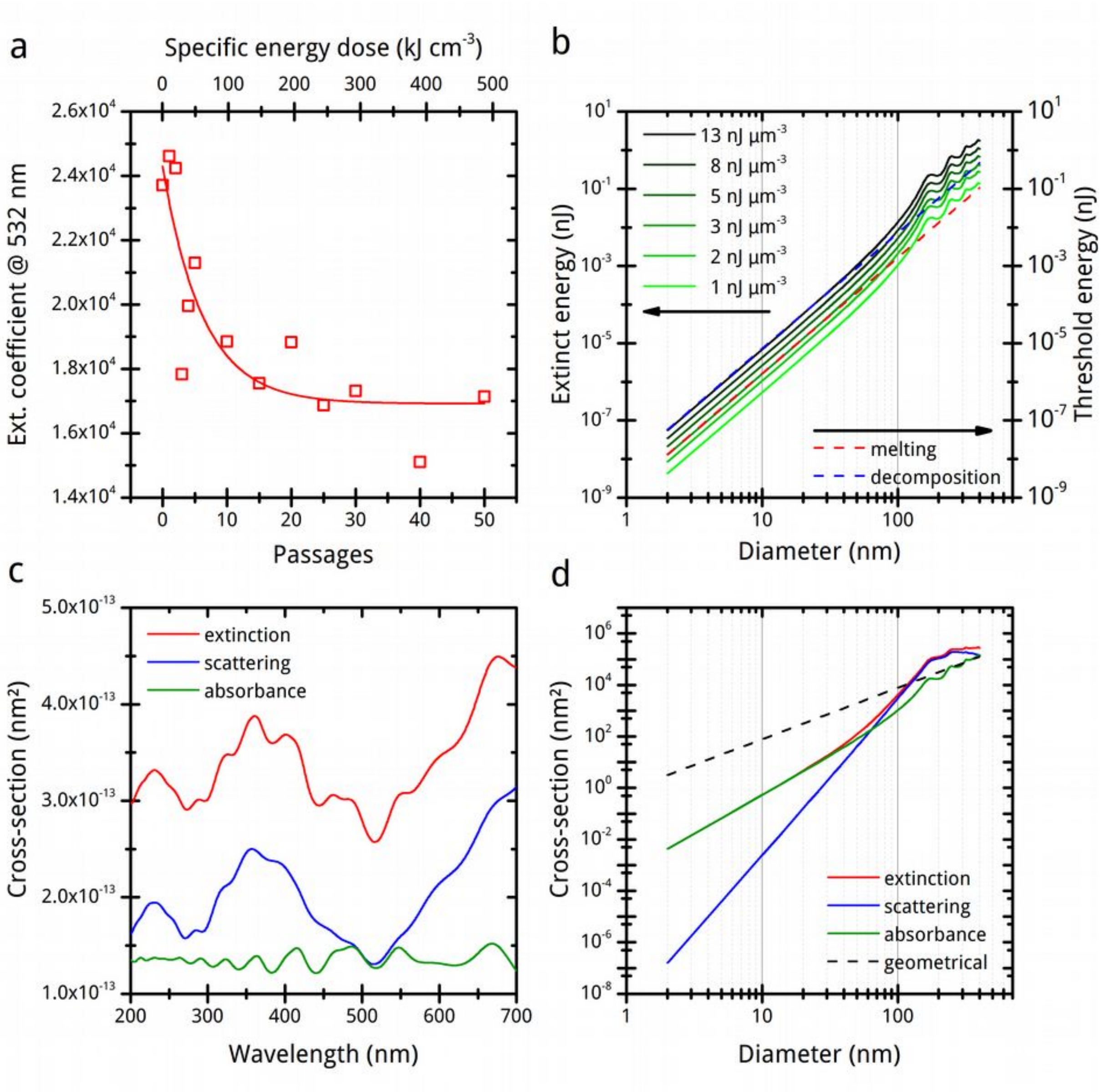

Supplementary Figure S7: Change of extinction coefficient of products of PLFL in dependence to their passage number (a), comparison of absorbed and threshold energy versus particle diameter for different energy density regimes in the liquid jet (b), optical cross-sections of a $CoFe_2O_4$ sphere of 200 nm diameter versus wavelength (c) and geometrical as well as optical cross-sections of $CoFe_2O_4$ spheres versus particle diameter (d).

Based on model data and particles Mie absorption cross-section[S13], we calculate the by single particles of different sizes absorbed energy in relation to the different energy densities existing in the liquid jet at applied experimental conditions. Real and imaginary part of $CoFe_2O_4$'s refractive index, we calculate on the complex permittivity data for a 69 nm thick $CoFe_2O_4$ thin film[S14]. Since the indices lead to a much too high absorption coefficient, we correct them to fit the extinction measured by UV-vis spectroscopy. Of course, extinction is divided into

absorption and scattering. Thus, we do a rough estimation here. Furthermore, we compare the absorbed energy of single particles to the specific energies needed for thermal melting and decomposition processes of whole the particle mass. Specific enthalpies for $CoFe_2O_4$ cannot be found, but for highly comparable Fe_3O_4[S15]. The decomposition of Fe_3O_4 to Fe takes place stepwise with an intermediate at FeO. Specific enthalpies of both steps are comparable, 2,613 J g^{-1} (Fe_3O_4 to FeO) and 2,287 J g^{-1} (FeO to Fe). The diagram in Supplementary Fig. S7b shows the comparison of absorbed energy by single particles in dependence to their diameter for different energy density regimes in the liquid jet. The threshold line for the decomposition corresponds to that of Fe_3O_4 to FeO since the needed energy is a bit higher than for FeO to Fe. For interesting particle sizes, we illustrate the state of particle transformation in dependence to its position within the liquid jet during one irradiating laser pulse in Fig. 8c-f. Whereas melting and decomposition regimes are colored, areas of no possible full transformations are white. The black circle marks the jet border. Melting, and conclusively fusion due magnetic agglomeration, is the predominant mechanism for all particle diameters. The diagram in Supplementary Fig. S7c is supplementary presenting the different cross-sections, geometrical and optical, versus the particle diameter. It clearly shows that absorption is dominating scattering up to a diameter 60 nm. At higher diameters, the contribution of scattering to the extinction is predominant. However, we can conclude that a thermal decomposition of full particles is unlikely and only taking place for particles of higher diameters. Thermal melting of entire particles, on the other hand, is very probable for bigger particles and can also affect the smallest observed particles. Our model results are limited and need further refinements to fit the real conditions during the laser irradiation of a $CoFe_2O_4$ nanocolloid. However, the model already predicts the existence of different thermal processes, which products can be find by our applied analytics.

We show an overview of the different products generated from the $CoFe_2O_4$ educt during PLFL in water in Supplementary Fig. S8. At the top level, we start with the educt particles in

the mid which can be partly (to the left) or fully (to the right) melted depending on the absorbed laser energy. Moreover, bigger educt particles completely decompose during high laser energy absorption. Products of this decomposition process (in the mid of the second level) are ultra-small, amorphous particles and Fe oxyhydroxide species. We expect the ultra-small particles to have a shift in stoichiometry away from $CoFe_2O_4$ towards a Co-richness since fully (to the right) melted products contain crystalline CoO. Partly melted (to the left) species at this level are amorphous like the ultra-small particles. At the bottom level, we find a byproduct of the decomposition which most likely forms during laser irradiation of solved Fe oxyhydroxides.

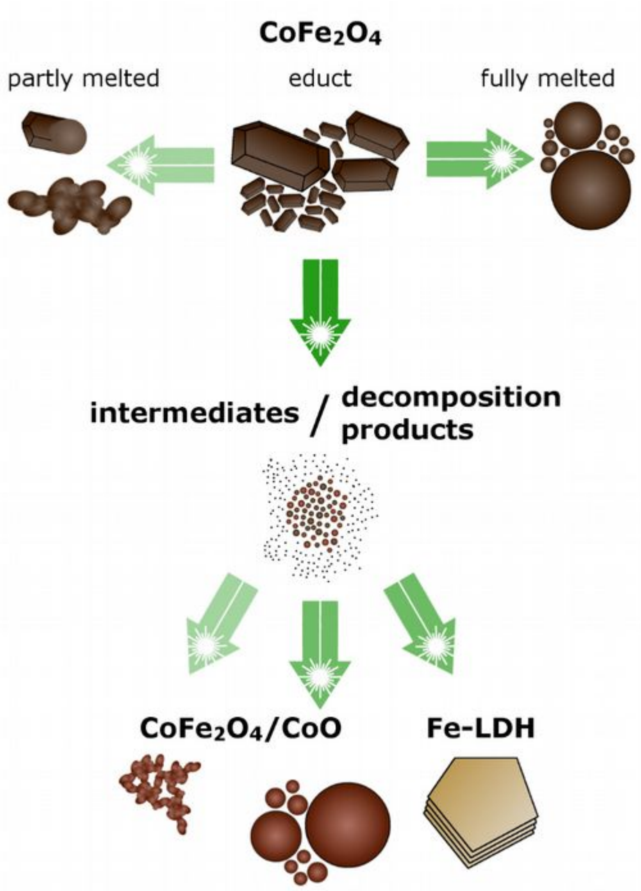

Supplementary Figure S8: Schematic cartoon of laser-induced product generation from $CoFe_2O_4$ educt during PLFL. $CoFe_2O_4$ species (top level) include the educt and laser-melted species. Laser-decomposition results in ultra-small, amorphous particles and solved oxyhydroxides (middle level). Further laser irradiation of decomposition products leads to crystalline $CoFe_2O_4$/CoO particles and amorphous network structures due to melting and Fe-LDH sheets.

References

S1 Brunauer, S., Emmett, P. H. & Teller, E. Adsorption of gases in multimolecular layers. *J. Am. Chem. Soc.* ***60***, 309-319 (*1938)*.

S2 Ok, H. N. & Mullen, J. G. Evidence of two forms of cobaltous oxide. *Phys. Rev.* **168**, 550 (1968).

S3 Jay, A. H. & Andrews, K. W. Note on oxide systems pertaining to steel-making furnace slags-FeO-MnO, FeO-MgO, CaO-MnO, MgO-MnO. *J. Iron Steel I.* **152**, 15-18 (1945).

S4 Rieck, G. D. & Thijssen, J. J. M. The cation distribution in $CoFe_2O_4$. *Acta. Crystallogr. Sect. B* **24**, 982-983 (1968).

S5 Van der Gaast, S. J. & Vaars, A. J. Method to eliminate the background in X-ray diffraction patterns of oriented clay mineral samples. *Clay Miner.* **16**, 383-393 (1981).

S6 Will, G., Masciocchi, N., Parrish, W. & Hart, M. *J. Appl. Crystallogr.* **20**, 394-401 (1987).

S7 Ruby, C. *et al*. Synthesis and transformation of iron-based layered double hydroxides. *Appl. Clay. Sci.* **48**, 195-202 (2010).

S8 Ishikawa, Y., Koshizaki, N. & Pyatenko, A. Submicrometer-sized spherical iron oxide particles fabricated by pulsed laser melting in liquid. *Electron. Comm. Jpn.* **99**, 37-42 (2016).

S9 He, J. *et al*. Preparation of layered double hydroxides. In *Layered double hydroxides* (eds. Evans, D. G. & Duan, X.) 89-119 (Springer, 2006).

S10 Hunter, B. M. *et al*. Highly active mixed-metal nanosheet water oxidation catalysts made by pulsed-laser ablation in liquids. *J. Am. Chem. Soc.* **136**, 13118-13121 (2014).

S11 Refait, P., Charton, A. & Génin, J. M. Identification, composition, thermodynamic and structural properties of a pyroaurite-like iron (II)-iron (III) hydroxy-oxalate green rust. *Eur. J. Inorg. Chem.* **35**, 655-666 (1998).

S12 Hale, G. M. & Querry, M. R. Optical constants of water in the 200 nm to 200 μm wavelength region. *Appl. Opt.* **12**, 555-563 (1973).

S13 Mie, G. Beiträge zur Optik trüber Medien, speziell kolloidaler Metallösungen. *Ann. Phys.* **330**, 377-445 (1908). German.

S14 Himcinschi, C. *et al*. Optical and magneto-optical study of nickel and cobalt ferrite epitaxial thin films and submicron structures. *J. Appl. Phys.* **113**, 084101 (2013).

S15 *The oxide handbook* (ed. Samsonov, G. V.) (Springer, 2013).
